# SARS-CoV-2 infection, inflammation and birth outcomes in a prospective NYC pregnancy cohort

**DOI:** 10.1016/j.jri.2024.104243

**Published:** 2024-03-18

**Authors:** Frederieke A.J. Gigase, Rebecca H. Jessel, Elianna Kaplowitz, Natalie Boychuk, Sophie Ohrn, Erona Ibroci, Juliana Castro, Jezelle Lynch, Rushna Tubassum, Amy Balbierz, Nina M. Molenaar, Mara Graziani, Roy Missall, Tammy Flores, Toni Stern, Juan Manuel Carreno, Krammer Serology Core Study Group, Florian Krammer, Alan Adler, Rachel I. Brody, Corina Lesseur, Jia Chen, Sascha Ellington, Romeo R. Galang, Margaret C. Snead, Elizabeth Howell, Joanne Stone, Veerle Bergink, Siobhan Dolan, Whitney Lieb, Anna-Sophie Rommel, Lotje D. de Witte, Teresa Janevic

**Affiliations:** a Department of Psychiatry, Icahn School of Medicine at Mount Sinai, New York City, NY, USA; b Department of Obstetrics, Gynecology and Reproductive Science, Icahn School of Medicine at Mount Sinai, New York City, NY, USA; c Blavatnik Family Women’s Health Research Institute, Icahn School of Medicine at Mount Sinai, New York City, NY, USA; d Department of Population Health Science and Policy, Icahn School of Medicine at Mount Sinai, New York City, NY, USA; e Department of Pathology, Molecular and Cell Based Medicine, Icahn School of Medicine at Mount Sinai, New York City, NY, USA; f Department of Obstetrics and Gynecology, Perelman School of Medicine, University of Pennsylvania, Philadelphia, PA, USA; g Department of Environmental Medicine and Public Health, Icahn School of Medicine at Mount Sinai, New York City, NY, USA; h Department of Child and Adolescent Psychiatry, Erasmus Medical Center, Rotterdam, the Netherlands; i COVID-19 Response, Centers for Disease Control and Prevention, Atlanta, GA, USA; j Department of Microbiology, Icahn School of Medicine at Mount Sinai, New York, NY, USA; k Center for Vaccine Research and Pandemic Preparedness (C-VaRPP), Icahn School of Medicine at Mount Sinai, New York, NY, USA; l Department of Psychiatry, Erasmus Medical Center, Rotterdam, the Netherlands

**Keywords:** SARS-CoV-2, Inflammation, Cytokines, HS-CRP, Preterm birth, Birthweight

## Abstract

Associations between antenatal SARS-CoV-2 infection and pregnancy outcomes have been conflicting and the role of the immune system is currently unclear. This prospective cohort study investigated the interaction of antenatal SARS-CoV-2 infection, changes in cytokine and HS-CRP levels, birthweight and gestational age at birth. 2352 pregnant participants from New York City (2020–2022) were included. Plasma levels of interleukin (IL)-1β, IL-6, IL-17A and high-sensitivity C-reactive protein (HS-CRP) were quantified in blood specimens obtained across pregnancy. Quantile and linear regression models were conducted to 1) assess the impact of antenatal SARS-CoV-2 infection, overall and by timing of detection of SARS-CoV-2 positivity (< 20 weeks versus ≥ 20 weeks), on birthweight and gestational age at delivery; 2) examine the relationship between SARS-CoV-2 infection and maternal immune changes during pregnancy. All models were adjusted for maternal demographic and obstetric factors and pandemic timing. Birthweight models were additionally adjusted for gestational age at delivery and fetal sex. Immune marker models were also adjusted for gestational age at specimen collection and multiplex assay batch. 371 (15.8%) participants were infected with SARS-CoV-2 during pregnancy, of which 98 (26.4%) were infected at < 20 weeks gestation. Neither SARS-CoV-2 infection in general nor in early or late pregnancy was associated with lower birthweight nor earlier gestational age at delivery. Further, we did not observe cytokine or HS-CRP changes in response to SARS-CoV-2 infection and thus found no evidence to support a potential association between immune dysregulation and the diversity in pregnancy outcomes following infection.

## 1. Introduction

As the severe acute respiratory syndrome coronavirus 2 (SARS-CoV-2) pandemic has progressed, antenatal infection has been reported to be associated with an array of adverse perinatal outcomes including preterm birth, preeclampsia, intensive care unit (ICU) admission, and maternal death (Cavalcante et al., 2021; Egloff et al., 2023; E. R. Smith et al., 2023). However, the majority of these findings are in the setting of at least moderate maternal illness (Brandt et al., 2021; Metz et al., 2022). Data about pregnancy outcomes following asymptomatic SARS-CoV-2 infection is limited and fail to demonstrate an increased risk of preterm birth nor low birthweight (Molenaar et al., 2022; L. H. Smith et al., 2022). Similarly, studies found no association between early pregnancy SARS-CoV-2 infection and preterm birth or small for gestational age (Fallach et al., 2022; L. H. Smith et al., 2022). However, given limitations in prior study design, including (1) varying control groups of both pregnant and non-pregnant individuals, (2) recruitment in late pregnancy or at delivery and (3) recruitment of hospitalized and/or severe SARS-CoV-2 cases based on positive RT-PCR, the impact of early pregnancy or less severe SARS-CoV-2 infection remains unclear (Brandt et al., 2021; Metz et al., 2022; L. H. Smith et al., 2022). Therefore, unbiased sampling in a large prospective pregnancy cohort is crucial.

SARS-CoV-2 infection during pregnancy has been associated with increased levels of interleukin IL-2, IL-6, IL-8, IL-10 and IL-17 after first trimester SARS-CoV-2 infection and interferon (IFN)-γ after third trimester SARS-CoV-2 infection compared to non-infected pregnant women in plasma collected upon admission to the hospital or at delivery, in severe immune activation also referred to as the ‘cytokine storm’ (Cavalcante et al., 2021; Egloff et al., 2023; Taglauer et al., 2022; Tanacan et al., 2021). However, others found no difference in cytokine levels between SARS-CoV-2 infected cases and controls in plasma collected throughout gestation or upon admission to the hospital (Sherer et al., 2021; Trilla et al., 2022). Most previous studies are limited by small cohort size, inconsistent method of SARS-CoV-2 diagnosis, and variation in timing of specimen collection for cytokine assessment. To date, the largest cohort, which included 180 pregnant patients, failed to demonstrate any difference in IL-6 between SARS-CoV-2 infected and non-infected pregnant women (Tanacan et al., 2021) (Supplementary Table 1).

The Generation C cohort represents a large diverse prospective cohort of pregnant persons recruited in one of the pandemic epicenters: New York City (NYC). Interim analyses on the effect of SARS-CoV-2 infection on various pregnancy outcomes in this cohort failed to reveal any associations (Janevic et al., 2022; Molenaar et al., 2022). In the current study, we (1) investigated the effect of antenatal SARS-CoV-2 infection on gestational age at delivery and birthweight in the entire cohort of more than 2000 patients. Moreover, we investigated if (2) SARS-CoV-2 infection resulted in changes in maternal cytokines and HS-CRP in early and late gestation, and if so (3) whether dysregulation of these markers is associated with adverse lower birthweight or earlier gestational age at birth.

## 2. Materials and methods

### 2.1. Study design and population

**Fig. 1.**
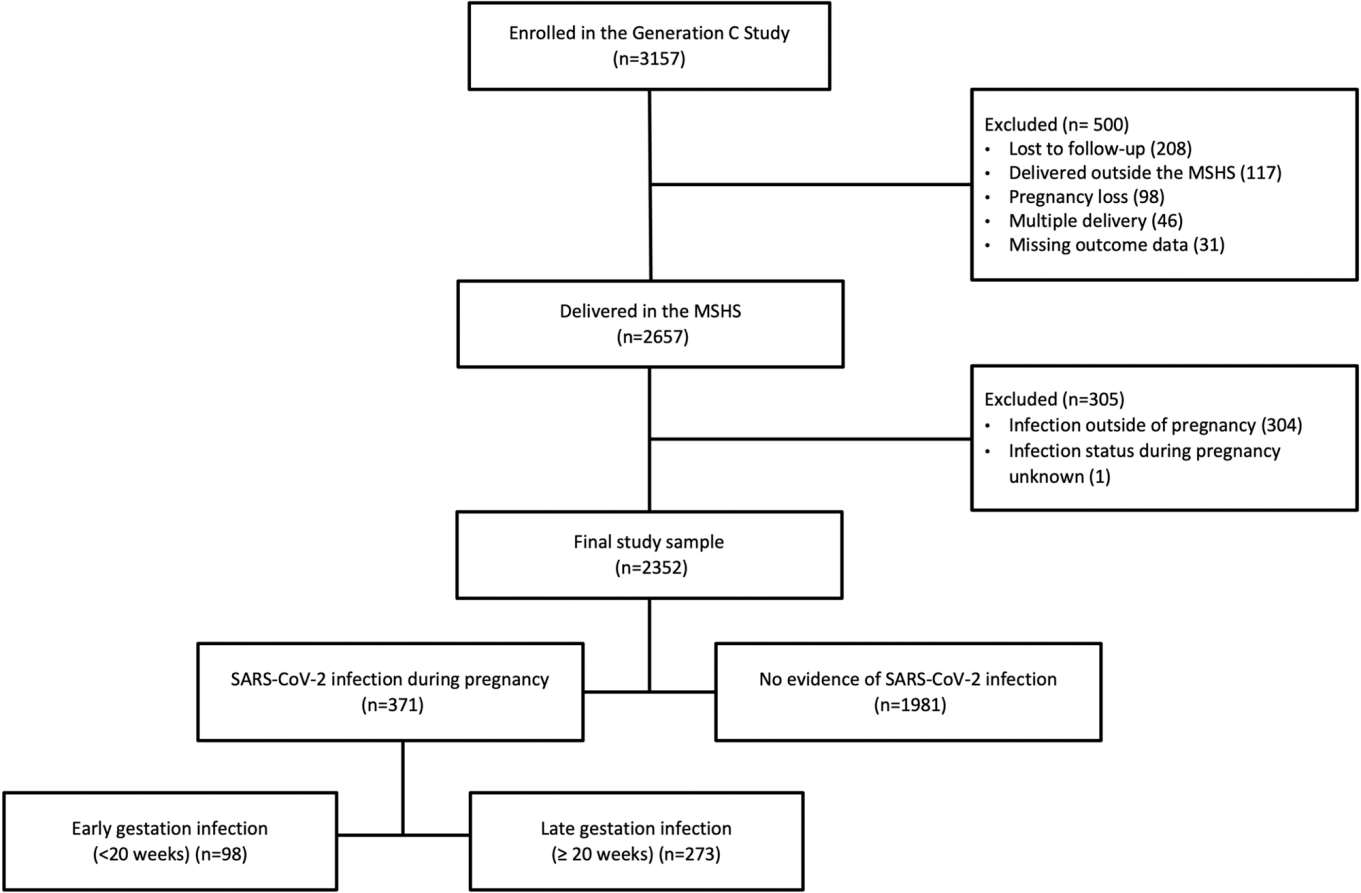
Flowchart of study sample.

The Generation C Study is a prospective cohort study conducted within the Mount Sinai Health System (MSHS), described elsewhere (Molenaar et al., 2022). Pregnant individuals (≥ 18 years old) who received obstetrical care in the MSHS were eligible for participation. Recruitment began in April 2020 and concluded in February 2022. All participants provided informed consent. The study was approved by the Icahn School of Medicine at Mount Sinai Institutional Review Board (IRB-20–03352), reviewed by the US Centers for Disease Control and Prevention (CDC), and consistent with applicable federal law and CDC policy. Exclusion criteria were incomplete data (lost to follow-up and missing outcome data), delivery outside of the MSHS, pregnancy loss, multiple gestation (due to lower birthweight and gestational age inherent to multiple gestation), or unknown infection status/infection outside of pregnancy (n = 805). The final cohort included 2352 pregnant participants (Fig. 1). All patient demographic and clinical data were extracted from the electronic medical record (EMR).

### 2.2. Specimen collection and processing

Blood specimens were obtained as part of routine blood draws (an additional 4cc EDTA tube) during scheduled prenatal visits or on admission to labor and delivery (median gestational age at plasma collection: 34.4 weeks, range = 4.9 – 42.1 weeks). Specimens were processed within 4 hours (median = 1.6 hours; IQR = 1.7 hours). Plasma aliquots were centrifuged, aliquoted into 500 μl vials and stored at −80 °C until further analysis.

### 2.3. Serological testing

Serological testing of plasma specimens was performed for every participant using a serologic enzyme-linked immunosorbent assay (ELISA) developed at the Icahn School of Medicine at Mount Sinai (Stadlbauer et al., 2020). Participants with anti-spike immunoglobulin G (IgG) positive specimens collected after the roll-out of COVID-19 vaccines on December 14, 2020, were further analyzed to determine the presence of anti-nucleocapsid IgG antibodies using the MILLIPLEX^®^ SARS-CoV-2 Antigen Panel 1 IgG from Millipore to confirm that antibodies were due to natural infection (Young et al., 2020).

### 2.4. SARS-CoV-2 infection status and timing

Participant SARS-CoV-2 infection status and exposure date was ascertained through 1) a positive RT-PCR test result during pregnancy; the date of the positive test was considered as the date of detection of SARS-CoV-2 positivity (n=161); 2) anti-S IgG antibody presence AND one of the following: a) anti-S IgG antibody before an individual’s first COVID-19 vaccination, b) anti-S IgG antibody before the COVID-19 vaccination rollout in NYC (Dec 14, 2020), or c) anti-spike IgG antibody presence and anti-N IgG antibody; the date the specimen was collected was considered as the date of detection of SARS-CoV-2 positivity (n=281); or 3) diagnosis by a medical health official reported in the EMR and/or questionnaire during pregnancy; the date of the EMR report was considered the date of detection of SARS-CoV-2 positivity (n=14). There is overlap between categories as participants may have multiple identification methods (e.g. both RT-PCR and anti-S IgG). Pregnant people were considered never infected if they had no indication of SARS-CoV-2 infection according to the determinants listed above.

### 2.5. Multiplex analysis of cytokines and HS-CRP

In 200 participants, a High Sensitivity T-cell Discovery Array 14-plex analysis was performed (Supplementary Figure 1 A and B). Based on the hierarchical clustering analysis described previously (Gigase et al., 2022), IL-1β, IL-6 and IL-17A were selected together with high sensitivity C-reactive protein (HS-CRP) as an established marker of general inflammation. Cytokines and HS-CRP were assessed in the entire cohort using the High Sensitivity T-cell Discovery Array 3-Plex (Millipore, St. Charles, MO, USA) at Eve Technologies using the Bio-Plex^™^ 200 system (Bio-Rad Laboratories, Inc., Hercules, CA, USA). Participants with missing cytokine values were excluded from analysis. For post-hoc analyses, HS-CRP was divided into a normal HS-CRP group (HS-CRP < 40 mg/L) and a high HS-CRP group (HS-CRP ≥ 40 mg/L), based on the distribution of HS-CRP in the current sample (90th percentile: 40 mg/L). If multiple specimens were collected from the same participant, the specimen collected closest to SARS-CoV-2 infection was used for analyses. Each specimen of infected participants was matched to one non-infected specimens based on gestational age (+/− 1 week) to control for known cytokine variation in advancing pregnancy. Infected participants were excluded from these analyses if infection occurred after specimen collection or if no specimen was collected (excluded n=86). Never infected participants were excluded if they did not have a specimen that could be matched (n=165). A total of 2101 participants were included in the final immune marker analyses.

### 2.6. Statistical analysis

Demographics and pregnancy outcomes were compared between never infected, early and late infection groups using T-test, Wilcoxon Rank Sum test, ANOVA or Kruskal-Wallis test, Chi-square or Fisher’s exact test where appropriate. The associations between SARS-CoV-2 infection (any, early or late) and birthweight and gestational age at delivery were assessed using multiple univariate and multivariate quantile regression models for the 25%, 50%, and 75% quantiles. This modeling approach has the advantage of examining differences across the distribution of birthweight/gestational age at delivery. The associations between early pregnancy SARS-CoV-2 infection and early and late gestation IL-1β, IL-6, IL-17A and HS-CRP and between late gestation SARS-CoV-2 infection and late gestation IL-1β, IL-6, IL-17A and HS-CRP were assessed using multiple linear regression analyses. The associations between early and late gestation IL-1β, IL-6, IL-17A and HS-CRP and birthweight and gestational age at delivery were assessed with univariate and multivariate quantile regression models for the 25%, 50%, and 75% quantiles. A post-hoc multiple logistic regression analysis was performed to assess the impact of SARS-CoV-2 infection (any, early or late) on high HS-CRP (≥ 40 mg/L). Post-hoc univariate and multivariate quantile regression models for the 25%, 50%, and 75% quantile to investigate the association between early and late gestation high HS-CRP and birthweight and gestational age at delivery. We repeated several analyses in a sensitivity analysis of infected participants only to investigate whether associations were different after infection. Cytokine and HS-CRP values were log2 transformed for normalization purposes and analyzed as continuous variables. All multivariable quantile and linear regression models were adjusted for the covariates listed below. Analyses were conducted using SPSS 28.0 (IBM SPSS Statistics).

### 2.7. Covariates

Covariates were chosen a priori based on existing literature and included maternal age categorized by advanced maternal age (AMA) status (<34 years, ≥35 years), self-reported race/ethnicity (Hispanic, Asian (non-Hispanic), Black (non-Hispanic), White (non-Hispanic), any other race), insurance (private/self-pay, public), parity, pre-pregnancy BMI (underweight/normal weight (<18.5–24.9), overweight (25.0–29.9), obese (>30)), history of preterm birth (yes, no), chronic hypertension (yes, no), pre-existing diabetes (yes, no), gestational hypertension (yes, no), gestational diabetes (yes, no), pre-eclampsia (yes, no), COVID-19 vaccination status (at least one dose of any vaccine type: yes during pregnancy, yes prior to pregnancy, no/unknown), and time between the first confirmed case of SARS-CoV-2 in NYC (March 1, 2020) and birth (weeks) in order to control for different regulations and virus variants (Deng et al., 2022). In models evaluating birthweight as outcome, we additionally adjusted for gestational age at delivery and fetal sex. In immune marker models, we additionally adjusted for gestational age at specimen collection and multiplex assay batch.

## 3. Results

### 3.1. Characteristics of the cohort

Of 2352 participants, 371 (15.8%) participants had antenatal SARS-CoV-2 infection, of which 98 (26.4%) were infected at < 20 weeks gestation, and 273 (73.6%) were infected at ≥ 20 weeks gestation (Fig. 1). Five participants 0.2% were hospitalized for management of their SARS-CoV-2 illness. The median age of participants was 33 years (range 18–49 years). Infected individuals were younger, more likely to be Hispanic or Black, publicly insured, and obese relative to those who were not infected (Table 1). Median gestational age at delivery was 39.1 weeks (range between 24.4 and 42.1 weeks), and median birthweight was 3250 g (range between 530.1 and 5749.9 g).

### 3.2. SARS-CoV-2 infection and birthweight and gestational age at delivery

**Fig. 2.**
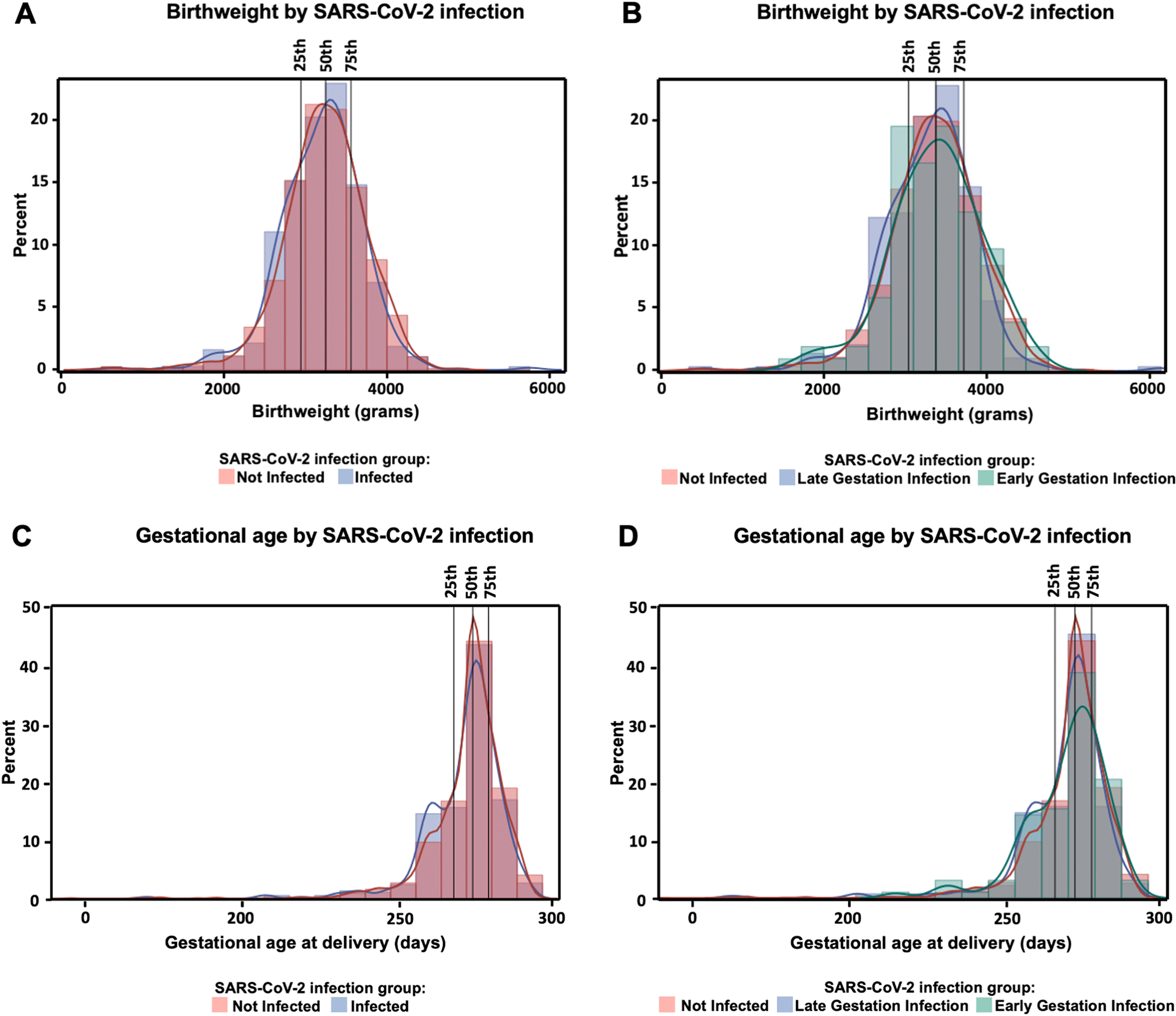
Multivariate quantile regression of SARS-CoV-2 infection on birthweight (grams) and gestational age (GA) at delivery (days). A) Birthweight (grams) compared between infected and not infected participants. Results indicate no significant difference in birthweight between groups. B) Birthweight (grams) compared between early gestation infection (<20 weeks), late gestation infection (≥20 weeks) and not infected participants. Results indicate increased birthweight at the 50% quantile after early gestation SARS-CoV-2 infection. C) GA at delivery (days) compared between infected and not infected participants. Results indicate no significant difference in GA at delivery between groups. D) GA at delivery (days) compared between early gestation infection (<20 weeks), late gestation infection (≥20 weeks) and not infected participants. Results indicate no significant difference in GA at delivery between groups. Analyses were adjusted for confounders.

Pregnancy outcomes were not significantly different between SARS-CoV-2 infected participants and non-infected pregnant controls (Table 1). Birthweight and gestational age at delivery were investigated further in multivariable analyses. After adjusting for covariates, participants with early infection (<20 weeks gestation) delivered neonates weighing 103.96 g more than neonates of non-infected participants (*p*=0.044 at 50% quantile; Supplementary Table 2; Fig. 2B). There was no association between *any* antenatal SARS-CoV-2 infection with birthweight or gestational age at delivery adjusted analyses (*p*>0.05 at 25%, 50% and 75% quantiles) (Supplementary Table 2; Fig. 2A, C and D). Findings were not different based on mode of SARS-CoV-2 status identification (e.g. RT-PCR or anti-S IgG measurement) (Supplementary Table 3).

### 3.3. SARS-CoV-2 infection and the maternal immune response

**Fig. 3.**
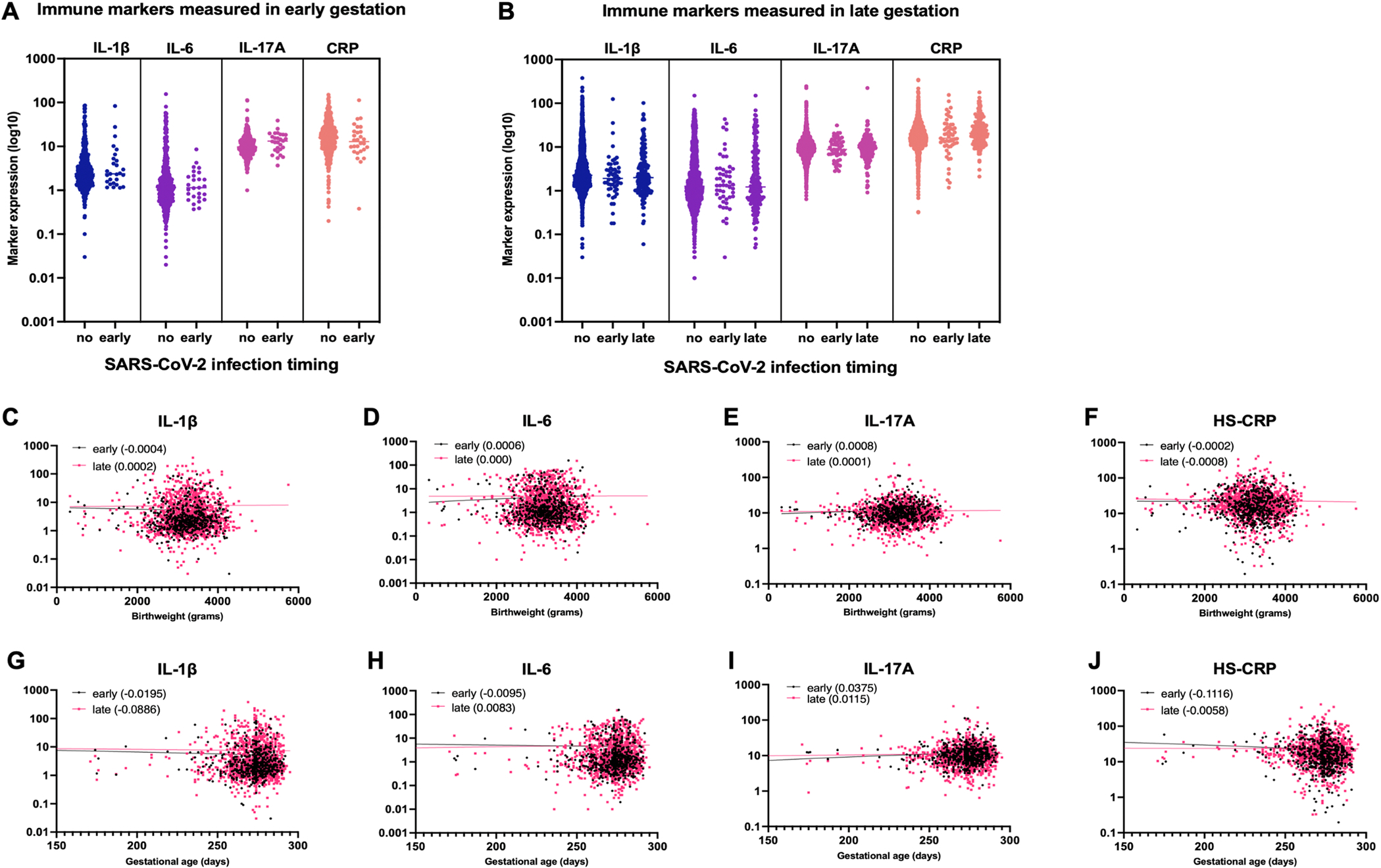
Levels of early (<20 weeks) and late (≥20 weeks) gestation IL-1β, IL-6, IL-17A, HS-CRP after SARS-CoV-2 infection (A-B) and the association with birthweight (C-F) and gestational age (GA) at delivery (G-J). Cytokine values (pg/ml) and HS-CRP (mg/L) are log10 transformed. A) Early gestation IL-1β, IL-6, IL-17A and HS-CRP (<20 weeks) in not infected participants (no) and participants with early gestation SARS-CoV-2 infection (early). B) Late gestation IL-1β, IL-6, IL-17A and HS-CRP (≥20 weeks) in not infected participants (no), participants with early gestation SARS-CoV-2 infection (early) and participants with late gestation SARS-CoV-2 infection (late). C-F) Early gestation IL-17A and late gestation IL-1β and HS-CRP were significantly associated with GA (days) after adjusting for confounders. The legend indicates the slope of the regression line for the early and late gestation IL-1β, IL-6, IL-17A and HS-CRP, respectively. G-J) Early and late gestation IL-1β, IL-6, IL-17A and HS-CRP were not significantly associated with birthweight after adjusting for confounders. The legend indicates the slope of the regression line for the early and late gestation IL-1β, IL-6, IL-17A and HS-CRP, respectively.

IL-1β, IL-6, IL-17A and HS-CRP were analyzed in specimens from 2101 participants (Supplementary Figure 1 C&D). The median number of weeks between SARS-CoV-2 infection and specimen collection was 5.4 weeks (range 0–35 weeks) for participants infected in early gestation and 0 weeks (range 0–18.4 weeks) for participants infected in late gestation. A sensitivity analysis of participants with specimens collected within 14 days (n=132) and 30 days (n=150) of SARS-CoV-2 infection, as well as within 14 days (n=20) and 30 days (n=29) of confirmed RT-PCR positivity yielded similar results to the full cohort. Of 2101 participants, 285 (13.6%) had antenatal SARS-CoV-2 infection (early gestation infection, n=84 (29.5%); late gestation infection, n=201 (70.5%). In adjusted analyses, SARS-CoV-2 infection at < 20 weeks gestation was not associated with any changes in IL-1β, IL-6, IL-17A and HS-CRP (Tables 2a 2b; Fig. 3A and B). Late gestation (≥ 20 weeks) infection was associated with an increase in HS-CRP (1.15 mg/L) compared to not infected participants (*p*=0.011) (Table 2c; Fig. 3B). Findings were not different based on mode of SARS-CoV-2 status identification (e.g. RT-PCR or anti-S IgG measurement).

### 3.4. Maternal immune response and birthweight and gestational age at delivery

In adjusted analyses, cytokines and HS-CRP levels were not associated with birthweight (Table 3). Early gestation elevated IL-17A levels were associated with increased gestational age at delivery at the 50% and 75% quantile, indicating an increase of 1.04 and 1.54 days, respectively, with every unit increase in IL-17A (*p*=0.038; *p*=0.004, Table 4). Late gestation elevated IL-1β and HS-CRP levels were associated with decreased gestational age at delivery at the 25% and 50% quantile, respectively, indicating a decrease of 0.5 days with every unit increase in IL-1β and HS-CRP (*p*=0.013; *p*=0.034, Table 4) (Fig. 3C–J). In a sensitivity analysis of SARS-CoV-2 infected participants, we found several associations between cytokine and HS-CRP levels and birthweight and gestational age at delivery. Early gestation elevated IL-6, IL-17A, and HS-CRP, and late gestation IL-17A, were associated with birthweight (Supplementary Table 4), and late gestation IL-1β was associated with lower gestational age at delivery (Supplementary Table 5).

### 3.5. High HS-CRP and gestational age at delivery and birthweight

In a post-hoc analysis, we found no association between SARS-CoV-2 infection in early and late gestation and high HS-CRP (Supplementary Table 6a–c). High HS-CRP <20 weeks or ≥ 20 weeks gestation was not associated with birthweight (Supplementary Table 7a; Supplementary Figure 2A and B). High HS-CRP ≥ 20 weeks gestation was significantly associated with lower gestational age at delivery at the 50% quantile (*β* = − 2.65, *p*<0.001) and the 75% quantile (*β* = − 2.12 days, *p*<0.05; Supplementary Table 7b; Supplementary Figure 2C and D). When we repeated this analysis in SARS-CoV-2 infected participants only, high HS-CRP ≥ 20 weeks gestation was significantly associated with lower gestational age at delivery at the 25% quantile (*β* = −4.40 days, *p*<0.01) and the 50% quantile (*β* = −3.49 days, *p*<0.01; Supplementary table 8a and b).

## 4. Discussion

### 4.1. Principal findings

The current study did not find clinically significant associations between antenatal SARS-CoV-2 infection and birthweight or gestational age at delivery, nor cytokines and HS-CRP in early and late pregnancy after SARS-CoV-2 infection. With the exception of IL-1β and HS-CRP, cytokines were not associated with birthweight and gestational age at delivery. Our data confirm that high HS-CRP, regardless of SARS-CoV-2 infection, is associated with a significantly lower gestational age at delivery, which is consistent with a long-held hypothesis that preterm birth is related to inflammation (Romero et al., 2006).

### 4.2. Results in the context of what is known

The prevalence of antenatal SARS-CoV-2 infection in the current study (15.8%) is similar to the interim study of the same cohort (Molenaar et al., 2022) (16.4%) and higher compared to other pregnancy cohorts (e.g., 6.5% (Fallach et al., 2022); 2.5% (Balachandren et al., 2022); 3.2% (Darling et al., 2023)). Our findings concur with previous cohort studies including asymptomatic and first trimester-infected participants, which showed no association between SARS-CoV-2 infection and birthweight and preterm birth (Cosma et al., 2022; Crovetto et al., 2021; la Cour Freiesleben et al., 2021; E. R. Smith et al., 2023; Tanacan et al., 2021). Further, in a large cohort study by Metz et al., adverse perinatal outcomes were more frequent among patients with more severe illness (Metz et al., 2022). The idea that asymptomatic or mild SARS-CoV-2 infection in the second half of pregnancy is not associated with increased risk of preterm delivery, lower birthweight or reduced gestational age at delivery is further supported by previous work (L. H. Smith et al., 2022).

Previous studies on maternal cytokine levels in pregnant women with SARS-CoV-2 infection have reported conflicting results, possibly due to variations in study design and factors that may influence cytokine levels such as the timing of sample collection and severity of disease (Egloff et al., 2023; Rosen et al., 2022; Taglauer et al., 2022; Tanacan et al., 2021). Studies of cytokine levels in relation to disease severity have reported inconsistent results. Garcia-Flores et. al. did not find an association between cytokine changes and disease severity (Garcia-Flores et al., 2022). Rosen et al., showed increased IL-18, IL-1Ra and IL-2Ra with more severe disease (Rosen et al., 2022). Tanacan et. al. described significant positive (IFN-*y* and IL-6) and negative (IL-2 and IL-10) correlations relative to disease severity (Tanacan et al., 2021). In the current study, we found no evidence of exacerbated cytokine response in the absence of severe cases. Cytokine assessment is dependent on timing of infection and gestational age (Mor and Cardenas, 2010), and it is difficult to compare absolute values as study designs, sample collection and processing times differ across studies. Thus, trends are considered more elucidative. Various studies assessed the impact of the acute immune response by measuring cytokine levels directly after SARS-CoV-2 infection (Garcia-Flores et al., 2022; Rubio et al., 2022; Trilla et al., 2022). Studies investigating prolonged immune response have reported persistent elevated cytokine levels more than two weeks after SARS-CoV-2 infection (Sherer et al., 2021), as well as at delivery after SARS-CoV-2 infection (Rosen et al., 2022; Tanacan et al., 2021). Interestingly, a study by Taglauer and colleagues (2022) indicated persistent elevated levels of IL-6 and IL-8 at delivery after SARS-CoV-2 infection in early pregnancy, but not after infection in late pregnancy (Taglauer et al., 2022). A recent meta-analysis demonstrated no evidence of dysregulated cytokine levels following SARS-CoV-2 infection during pregnancy, except for CXCL-10 and IFN-*y* in a subset of participants with acute SARS-CoV-2 infection (Jain et al., 2023). These findings are in line with our results indicating no evidence for a prolonged cytokine response following SARS-CoV-2 infection. Future studies are needed to further investigate the complex interactions between the maternal immune response and SARS-CoV-2 infection.

### 4.3. Clinical implications

The current study did not detect an association between antenatal SARS-CoV-2 infection at any point during pregnancy with lower birthweight or gestational age at delivery in the absence of severe disease. This has important clinical implications with respect to patient counseling and antenatal surveillance.

### 4.4. Research implications

Our data supports the hypothesis that excessive maternal inflammation during pregnancy plays a role in the pathogenesis of preterm birth. The finding that a high HS-CRP at > 20 weeks gestation was associated with 2 days earlier delivery supports the hypothesis that preterm birth is the result of immune dysregulation (Wei et al., 2010). Similarly, Chen et al. (2022) reported that high CRP in the third trimester was associated with preterm birth ([aOR] = 1.49, 95% confidence interval: 1.02, 2.23, *p*= 0.04) (Y.-C. S. Chen et al., 2022). However, despite the prospective collection and large cohort, we had insufficient power to examine timing of infection and preterm birth (delivery < 37 weeks; n=198 (8.4%)). Future research may further elucidate this association.

### 4.5. Strengths and limitations

This is one of the largest study populations to date to investigate cytokine and HS-CRP levels after antenatal SARS-CoV-2 infection. Further, specimen selection was not based on the exposure nor outcome. Infection status was ascertained by various strategies which likely afforded the ability to capture asymptomatic cases in specimens with evidence of antibodies without recollection of symptoms. Limitations include limited knowledge of severity of maternal illness, and the absence of severe disease as very few participants were hospitalized for management of their viral illness. Additionally, limited knowledge of timing of infection was available for cases whose SARS-CoV-2 infection status was determined based on positive antibodies (n=281) and was estimated based on sampling date. This could have led to misclassification of the exposure. While the presence of IgG-type antibodies is a good indication that a person was exposed to the virus or vaccine (Al-Tamimi et al., 2023; Amellal et al., 2023), in the absence of PCR results a combined analysis of more rapidly generated and degenerated IgM could have helped to determine whether participants were recently infected. Despite adjusting for multiple other covariates that might influence cytokines and HS-CRP levels, there may be unmeasured causes within individuals masking true association between SARS-CoV-2 infection and IL-1β, IL-6, IL-17A and HS-CRP levels. While the current panel of cytokines and HS-CRP was carefully selected based on hierarchical clustering, it should be noted that these reflect only part of ongoing immune processes in response to SARS-CoV-2 infection. Lastly, the late gestation infection group was larger (>70%) compared to those who were exposed early in pregnancy (<30%). This is mostly due to the timing of recruitment secondary to limited prenatal visits during multiple lockdowns compared to asymptomatic infections found during mandatory testing on Labor & Delivery. As a result, cytokine changes may be due to the process of labor and delivery and might mask true differences.

### 4.6. Conclusions

In this large, diverse prospective pregnancy cohort, we did not find an association between antenatal SARS-CoV-2 infection and lower birthweight and gestational age at delivery. Further, as SARS-CoV-2 infection did not result in changes in pro-inflammatory cytokines and HS-CRP, our results provide no evidence to support a potential association between immune dysregulation and the diversity in pregnancy outcomes following SARS-CoV-2 infection observed in the literature.

## Supplementary Material

Suppl figure 1

Suppl figure 2

Suppl doc

## Figures and Tables

**Table 1:** Demographic and obstetric characteristics of participants enrolled in the Generation C study by timing of detection of SARS-CoV-2 positivity.

Characteristic	All (n=2352)	Early Gestation Infection (<20 weeks) (n=98)	Late Gestation Infection (≥20 weeks) (n=273)	Not Infected (n=1981)	P-value*

**Maternal age (years), n (%)**					**0.010**
18–34	1517 (64.5)	66 (67.3)	196 (71.8)	1255 (63.4)	
35–49	835 (35.5)	32 (32.7)	77 (28.2)	726 (36.6)	
**Race-Ethnicity, n (%)** **					**<0.001**
Asian	287 (12.2)	2 (2.0)	13 (4.8)	272 (13.7)	
Black or African American	329 (14.0)	23 (23.5)	53 (19.4)	253 (12.8)	
Hispanic	641 (27.2)	48 (49.0)	115 (42.1)	478 (24.1)	
White	987 (42.0)	22 (22.4)	75 (27.5)	890 (44.9)	
Other***	88 (3.7)	2 (2.0)	16 (5.9)	70 (3.5)	
**Insurance, n (%)**					**<0.001**
Private	1767 (75.1)	65 (66.3)	166 (60.8)	1536 (77.6)	
Public	580 (24.6)	33 (33.7)	106 (38.8)	441 (22.2)	
**Chronic Hypertension** **	130 (5.6)	7 (7.1)	16 (5.9)	107 (5.4)	0.758
**Pre-existing Diabetes** **	53 (2.3)	4 (4.1)	7 (2.6)	42 (2.1)	0.414
**Pre-pregnancy BMI (kg/m^3^), n (%)** ***					**<0.001**
Underweight/Normal (to 24.9)	1094 (46.5)	36 (36.8)	97 (35.5)	956 (48.3)	
Overweight (25.0–29.9)	685 (29.2)	20 (20.4)	68 (24.9)	560 (28.3)	
Obese (30 or higher)	531 (22.6)	42 (42.9)	103 (37.7)	421 (21.3)	
**Received COVID-19 Vaccination, n (%)**					**<0.001**
Yes, at least one dose during pregnancy	244 (10.4)	13 (13.3)	15 (5.5)	216 (10.9)	
Yes, all doses prior to pregnancy	116 (4.9)	17 (17.3)	31 (11.4)	68 (3.4)	
No proof of vaccination during pregnancy	1987 (84.5)	67 (68.4)	226 (82.8)	1694 (85.5)	
Timing of vaccination unknown	5 (0.2)	1 (1.0)	1 (0.4)	3 (0.2)	
***Obstetric history***					
**Parity, n (%)**					**<0.001**
Nulliparous	1232 (52.4)	42 (42.9)	106 (38.8)	1084 (54.7)	
Multiparous	1120 (47.6)	56 (57.1)	167 (61.2)	897 (45.3)	
**Previous preterm birth, n (%)**	147 (6.3)	12 (12.2)	21 (7.7)	114 (5.8)	**0.022**
**Previous C-section, n (%)** **	348 (14.8)	23 (23.5)	48 (17.6)	277 (14.0)	**0.01**
***Obstetric outcomes***					
**Gestational age at delivery (weeks), median (range)**	39.1 (24.4, 42.1)	39.1 (31.4, 41.6)	39.1 (24.4, 41.6)	39.1 (24.9, 42.1)	0.186
Preterm birth (< 37 weeks), n (%)	198 (8.4)	9 (9.2)	25 (9.2)	164 (8.3)	0.835
**Birthweight (g), median (range)**	3250.0	3255.0	3229.9	3255.1	0.118
	(530.1, 5749.9)	(1714.9, 4380.0)	(680.4, 5749.9)	(530.1, 4939.9)	
**Gestational Diabetes**	305 (13)	13 (13.3)	40 (14.7)	252 (12.7)	0.682
**Gestational Hypertension** **	259 (11)	6 (6.1)	29 (10.6)	224 (11.3)	0.273
**Any Pre-Eclampsia** ****	198 (8.4)	10 (10.2)	24 (8.8)	164 (8.3)	0.789
**Delivery mode, n (%)**					**0.002**
Vaginal	1506 (64.0)	47 (48.0)	191 (70.0)	1268 (64.0)	
C-Section	846 (36.0)	51 (52.0)	82 (30.0)	713 (36.0)	
Apgar score 5 min, median (range)	9 (1– 10)	9 (5– 9)	9 (3– 9)	9 (1– 10)	0.148
Length of neonatal hospital stay in days, median (range)	2 (1– 160)	2 (1– 41)	2 (1– 119)	2 (1– 160)	0.735
NICU admission	205 (8.7)	7 (7.1)	23 (8.4)	175 (8.8)	0.856

* P-values compare the following three groups: early gestation infection, late gestation infection, never infected. P-values are calculated using ANOVA or Kruskal- Wallis test for continuous variables and Chi-square or Fisher’s exact test for categorical variables. P-values in **bold** show statistically significant results.

** 49 (2.1%) patients are missing BMI data, 22 (0.9%) patients are missing previous C-section data, 20 (0.8%) are missing Race/Ethnicity data, 18 (0.8%) patients are missing gestational hypertension and gestational diabetes data, 18 (0.8%) patients are missing Pre-eclampsia data, 5 (0.2%) patients are missing Insurance data, 3 (0.1%) patients are missing Chronic Hypertension and Pre-existing Diabetes data.

*** Other includes “American Indian/ Alaska Native”, “Native Hawaiian/ Other Pacific Islander”, and “Other” race.

**** Any Pre-eclampsia includes superimposed, mild and moderate.

**Table 2a:** Univariate and multivariate linear regression of early gestation (<20 weeks) SARS-CoV-2 infection on early gestation IL-1β, IL-6, IL-17A and HS-CRP **.

	Early gestation (<20 weeks) IL-1β		Early gestation (<20 weeks) IL-6		Early gestation (<20 weeks) IL-17A		Early gestation (<20 weeks) HS-CRP	

**Infection group**	**Coefficient (95% CI)**	**P-Value**	**Coefficient (95% CI)**	**P-Value**	**Coefficient (95% CI)**	**P-Value**	**Coefficient (95% CI)**	**P-Value**
**Unadjusted Analysis**								
Early Gestation Infection (<20 weeks) (n=54)	−0.21 (−0.65; 0.22)	0.332	−0.23 (−0.77; 0.32)	0.415	−0.15 (−0.37; 0.07)	0.188	**0.52 (0.15; 0.89)**	**0.006**
**Adjusted Analysis** *								
Early Gestation Infection (<20 weeks) (n=54)	−0.05 (−0.59; 0.49)	0.852	−0.88 (−0.81; 0.63)	0.810	−0.01 (−0.28; 0.27)	0.977	0.12 (−0.27; 0.51)	0.554

Coefficients, CI and P-values in bold signify statistically significant results.

* Adjusted for maternal age (18– 34, 35– 49), race/ethnicity (Asian (non-Hispanic), Black (non-Hispanic), Hispanic, White (non-Hispanic), any other race), insurance (private/self-pay, public), parity (nulliparous, multiparous), pre-pregnancy BMI (underweight (<18.5) & normal weight (18.5–24.9), overweight (25.0–29.9), obese (>30)), vaccinated any one dose (yes during pregnancy, yes prior to pregnancy, no), history of preterm birth (yes, no), chronic hypertension (yes, no), pre-existing diabetes (yes, no), gestational hypertension (yes, no), gestational diabetes (yes, no), pre-eclampsia (yes, no), time since start of pandemic, multiplex assay batch, and gestational age at specimen collection.

** Sample size adjusted to n=547 based on specimen availability. Early pregnancy infection n=54 and a reference group of not infected participants (n=494).

**Table 2b:** Univariate and multivariate linear regression of early gestation (<20 weeks) SARS-CoV-2 infection on late gestation IL-1β, IL-6, IL-17A and HS-CRP **.

Infection group	Late gestation (≥20 weeks) IL-1β		Late gestation (≥20 weeks) IL-6		Late gestation (≥20 weeks) IL-17A		Late gestation (≥20 weeks) HS-CRP	
	Coefficient (95% CI)	P-Value	Coefficient (95% CI)	P-Value	Coefficient (95% CI)	P-Value	Coefficient (95% CI)	P-Value

**Unadjusted Analysis**								
Early Gestation Infection (<20 weeks) (n=30)	−0.36 (−0.98; 0.26)	0.255	−0.31 (−1.06; 0.44)	0.418	−0.01 (−0.32; 0.31)	0.967	−0.17 (−0.56; 0.22)	0.400

**Adjusted Analysis** *								
Early Gestation Infection (*<*20 weeks) (n=30)	0.10 (−0.51; 0.71)	0.757	0.01 (−0.75; 0.78)	0.971	0.01 (−0.30; 0.33)	0.929	−0.26 (−0.62; 0.11)	0.176

Coefficients, CI and P-values in bold signify statistically significant results.

* Adjusted for maternal age (18– 34, 35– 49), race/ethnicity (Asian (non-Hispanic), Black (non-Hispanic), Hispanic, White (non-Hispanic), any other race), insurance (private/self-pay, public), parity (nulliparous, multiparous), pre-pregnancy BMI (underweight (<18.5) & normal weight (18.5–24.9), overweight (25.0–29.9), obese (>30)), vaccinated any one dose (yes during pregnancy, yes prior to pregnancy, no), history of preterm birth (yes, no), chronic hypertension (yes, no), pre-existing diabetes (yes, no), gestational hypertension (yes, no), gestational diabetes (yes, no), pre-eclampsia (yes, no), time since start of pandemic, multiplex assay batch, and gestational age at specimen collection.

** Sample size adjusted to n=1354 based on specimen availability. Early pregnancy infection n=30 and a reference group of not infected participants (n=1324).

**Table 2c:** Univariate and multivariate linear regression of late gestation (≥20 weeks) SARS-CoV-2 infection on late gestation IL-1β, IL-6, IL-17A and HS-CRP **.

Infection group	Late gestation (>20 weeks) IL-1β		Late gestation (≥20 weeks) IL-6		Late gestation (≥20 weeks) IL-17A		Late gestation (≥20 weeks) HS-CRP	
	Coefficient (95% CI)	P-Value	Coefficient (95% CI)	P-Value	Coefficient (95% CI)	P-Value	Coefficient (95% CI)	P-Value

**Unadjusted Analysis**								
Late Gestation Infection (≥20 weeks) (n=201)	**−0.26 (−0.52; ¡0.01)**	**0.041**	0.08 (−0.23; 0.38)	0.614	−0.00 (−0.13; 0.13)	0.979	**0.41 (0.25; 0.57)**	**<0.001**

**Adjusted Analysis** *								
Late Gestation Infection (≥20 weeks) (n=201)	−0.09 (−0.34; 0.17)	0.512	0.19 (−0.13; 0.51)	0.237	0.10 (−0.36; 0.23)	0.152	**0.20 (0.05; 0.36)** ***	**0.011**

Coefficients, CI and P-values in bold signify statistically significant results.

* Adjusted for maternal age (18– 34, 35– 49), race/ethnicity (Asian (non-Hispanic), Black (non-Hispanic), Hispanic, White (non-Hispanic), any other race), insurance (private/self-pay, public), parity (nulliparous, multiparous), pre-pregnancy BMI (underweight (<18.5) & normal weight (18.5–24.9), overweight (25.0–29.9), obese (>30)), vaccinated any one dose (yes during pregnancy, yes prior to pregnancy, no), history of preterm birth (yes, no), chronic hypertension (yes, no), pre-existing diabetes (yes, no), gestational hypertension (yes, no), gestational diabetes (yes, no), pre-eclampsia (yes, no), time since start of pandemic, multiplex assay batch, and gestational age at specimen collection.

** Sample size adjusted to n=1525 based on specimen availability. Early pregnancy infection n=201 compared to a reference group of not infected participants (n=1324).

*** Note that values are log2 transformed. 0.20 indicates 1.15 mg/L increase in late gestation HS-CRP (2^0.20)

**Table 3:** Univariate and multivariate quantile regression of early and late gestation IL-1β, IL-6, IL-17A and HS-CRP on birthweight (grams), n=2101.

	Birthweight (grams) 25% Quantile		Birthweight (grams) Median: 50% Quantile		Birthweight (grams) 75% Quantile	

**Early Gestation (<20 weeks), n=547**	**Coefficient (95% CI)**	**P-Value**	**Coefficient (95% CI)**	**P-Value**	**Coefficient (95% CI)**	**P-Value**
**Unadjusted Analysis**						
IL-1*β*	6.53 (−36.00; 49.02)	0.764	17.30 (−15.68; 50.27)	0.303	8.37 (−28.33; 45.07)	0.654
IL-6	4.90 (−29.13; 38.93)	0.778	−4.70 (−31.13; 21.72)	0.727	2.68 (−26.02; 31.39)	0.854
IL-17A	55.78 (−24.64; 136.19)	0.174	31.45 (−34.10; 97.00)	0.346	19.93 (−51.04; 90.89)	0.581
HS-CRP	−10.85 (−59.16; 37.47)	0.659	3.79 (−32.56; 40.14)	0.838	−15.12 (−56.79; 26.56)	0.476
**Adjusted Analysis** *						
IL-1*β*	−4.58 (−33.16; 24.01)	0.753	−5.90 (−32.77; 20.97)	0.666	−13.95 (−47.57; 19.67)	0.415
IL-6	0.81 (−19.75; 21.38)	0.938	0.24 (−20.46; 20.94)	0.982	8.55 (−15.69; 32.80)	0.488
IL-17A	15.58 (−37.49; 68.65)	0.564	−11.55 (−62.52; 39.41)	0.656	−19.83 (−82.23; 42.58)	0.533
HS-CRP	25.79 (−12.44; 64.02)	0.186	−1.56 (−37.01; 33.88)	0.931	32.53 (−10.32; 75.38)	0.136
	**Birthweight (grams)** 25% Quantile		**Birthweight (grams)** Median: 50% Quantile		**Birthweight (grams)** 75% Quantile	
**Late Gestation (≥20 weeks), n=1554**	**Coefficient (95% CI)**	**P-Value**	**Coefficient (95% CI)**	**P-Value**	**Coefficient (95% CI)**	**P-Value**
**Unadjusted Analysis**						
IL-1*β*	−3.98 (−23.76; 15.80)	0.693	−4.87 (−22.28; 12.54)	0.583	5.97 (−14.05; 25.95)	0.558
IL-6	3.20 (−13.33; 19.73)	0.704	4.19 (−10.22; 18.59)	0.569	−10.30 (−26.73; 6.13)	0.219
IL-17A	13.00 (−25.75; 51.76)	0.510	−6.17 (−40.25; 27.91)	0.723	12.14 (−26.67; 50.95)	0.540
HS-CRP	**−32.41 (−61.75; −3.07)**	**0.030**	−18.15 (−45.50; 9.21)	0.193	−10.52 (−42.38; 21.34)	0.517
**Adjusted Analysis** *						
IL-1*β*	−4.66 (−19.30; 9.97)	0.532	2.46 (−11.84; 16.75)	0.736	−5.79 (−23.85; 12.27)	0.530
IL-6	−9.00 (−20.83; 2.84)	0.136	−2.29 (−13.86; 9.27)	0.697	−8.05 (−22.23; 6.14)	0.266
IL-17A	−2.83 (−31.62; 25.95)	0.847	−4.50 (−31.60; 22.60)	0.745	3.38 (−31.29; 38.06)	0.848
HS-CRP	−14.70 (−39.50; 10.09)	0.245	−18.96 (−41.89; 3.98)	0.105	−9.47 (−38.74; 19.79)	0.526

Coefficients, CI and P-values in bold signify statistically significant results. Cytokines and HS-CRP are log2 transformed and analyzed as continuous variables.

* Adjusted for maternal age (18– 34, 35– 49), race/ethnicity (Asian (non-Hispanic), Black (non-Hispanic), Hispanic, White (non-Hispanic), any other race), insurance (private/self-pay, public), parity (nulliparous, multiparous), pre-pregnancy BMI (underweight (<18.5) & normal weight (18.5–24.9), overweight (25.0–29.9), obese (>30)), vaccinated any one dose (yes during pregnancy, yes prior to pregnancy, no), history of preterm birth (yes, no), chronic hypertension (yes, no), pre-existing diabetes (yes, no), gestational hypertension (yes, no), gestational diabetes (yes, no), pre-eclampsia (yes, no), time since start of pandemic, gestational age at delivery, fetal sex, multiplex assay batch, and gestational age at specimen collection.

**Table 4:** Univariate and multivariate quantile regression of early and late gestation IL-1β, IL-6, IL-17A and HS-CRP on gestational age at delivery (days).

	Gestational age at delivery (days) 25% Quantile		Gestational age at delivery (days) Median: 50% Quantile		Gestational age at delivery (days) 75% Quantile	

**Early Gestation (<20 weeks), n=547**	**Coefficient (95% CI)**	**P-Value**	**Coefficient (95% CI)**	**P-Value**	**Coefficient (95% CI)**	**P-Value**
**Unadjusted Analysis**						
IL-1β	0.26 (−0.97; 1.49)	0.677	0.00 (−0.54; 0.54)	>0.999	0.41 (−0.23; 1.05)	0.204
IL-6	0.18 (−0.75; 1.10)	0.709	0.00 (−0.43; 0.43)	>0.999	0.25 (−0.23; 0.73	0.307
IL-17A	1.62 (−0.59; 3.84)	0.151	0.00 (−1.03; 1.03)	>0.999	1.24 (−0.05; 2.52)	0.059
HS-CRP	**−1.34 (−2.58; −0.10)**	**0.034**	−0.49 (−1.09; 0.10)	0.105	−0.71 (−1.44; 0.04)	0.062
**Adjusted Analysis** *						
IL-1β	0.29 (−0.50; 1.07)	0.475	−0.19 (−0.71; 0.33)	0.474	−0.04 (−0.59; 0.50)	0.873
IL-6	−0.30 (−0.94; 0.33)	0.348	−0.01 (−0.40; 0.38)	0.954	0.20 (−0.21; 0.60)	0.343
IL-17A	0.54 (−1.00; 2.08)	0.490	**1.04 (0.06; 2.02)**	**0.038**	**1.54 (0.49; 2.57)**	**0.004**
HS-CRP	−0.56 (−1.65; 0.53)	0.315	−0.43 (−1.16; 0.30)	0.244	0.10 (−0.62; 0.81)	0.793
	**Gestational age at delivery (days)** 25% Quantile		**Gestational age at delivery (days)** Median: 50% Quantile		**Gestational age at delivery (days)** 75% Quantile	
**Late Gestation (≥20 weeks), n=1554**	**Coefficient (95% CI)**	**P-Value**	**Coefficient (95% CI)**	**P-Value**	**Coefficient (95% CI)**	**P-Value**
**Unadjusted Analysis** IL-1β	0.00 (−0.59; 0.59)	>0.999	0.00 (−0.20; 0.20)	>0.999	0.00 (−0.33; 0.33)	>0.999
IL-6	**0.53 (0.05; 1.01)**	**0.030**	0.00 (−0.16; 0.16)	>0.999	0.00 (−0.28; 0.28	>0.999
IL-17A	0.00 (−1.17; 1.17)	>0.999	0.00 (−0.39; 0.39)	>0.999	0.00 (−0.65; 0.65)	>0.999
HS-CRP	**−1.40 (−2.28; ¡0.52)**	**0.002**	**−0.44 (−0.78; ¡0.10)**	**0.012**	0.00 (−0.52; 0.52)	>0.999
**Adjusted Analysis** *						
IL-1β	**−0.49 (−0.88; ¡0.11)**	**0.013**	−0.21 (−0.47; 0.06)	0.126	−0.16 (−0.48; 0.15)	0.302
IL-6	−0.03 (−0.36; 0.30)	0.851	0.05 (−0.16; 0.25)	0.668	−0.01 (−0.26; 0.23)	0.913
IL-17A	−0.20 (−0.97; 0.57)	0.607	0.02 (−0.47; 0.51)	0.939	−0.11 (−0.68; 0.47)	0.720
HS-CRP	−0.17 (−0.84; 0.50)	0.625	**−0.46 (−0.89; ¡0.03)**	**0.034**	−0.29 (−0.78; 0.21)	0.255

Coefficients, CI and P-values in bold signify statistically significant results. Cytokines and HS-CRP are log2 transformed and analyzed as continuous variables.

* Adjusted for maternal age (18– 34, 35– 49), race/ethnicity (Asian (non-Hispanic), Black (non-Hispanic), Hispanic, White (non-Hispanic), any other race), insurance (private/self-pay, public), parity (nulliparous, multiparous), pre-pregnancy BMI (underweight (<18.5) & normal weight (18.5–24.9), overweight (25.0–29.9), obese (>30)), vaccinated any one dose (yes during pregnancy, yes prior to pregnancy, no), history of preterm birth (yes, no), chronic hypertension (yes, no), pre-existing diabetes (yes, no), gestational hypertension (yes, no), gestational diabetes (yes, no), pre-eclampsia (yes, no), time since start of pandemic, multiplex assay batch, and gestational age at specimen collection.
