# Supplementary figures and images for: SARS-CoV-2 infection, inflammation and birth outcomes in a prospective NYC pregnancy cohort
Source: J Reprod Immunol. Author manuscript; Available in PMC 2024 Jun 2. (PMC11144074; doi:10.1016/j.jri.2024.104243)

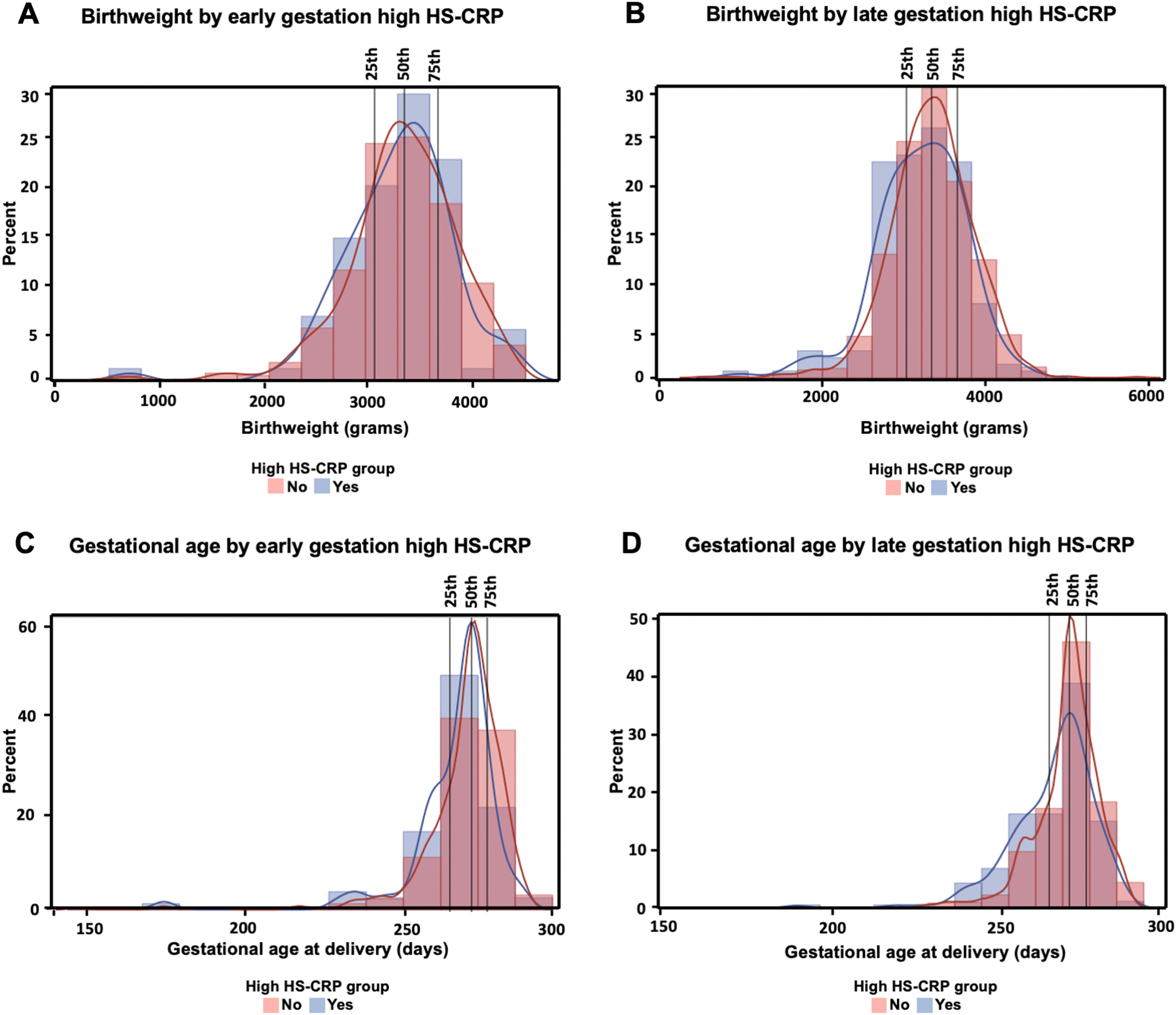

Supplement: Suppl figure 1 [file NIHMS1987502-supplement-Suppl_figure_1.jpg]

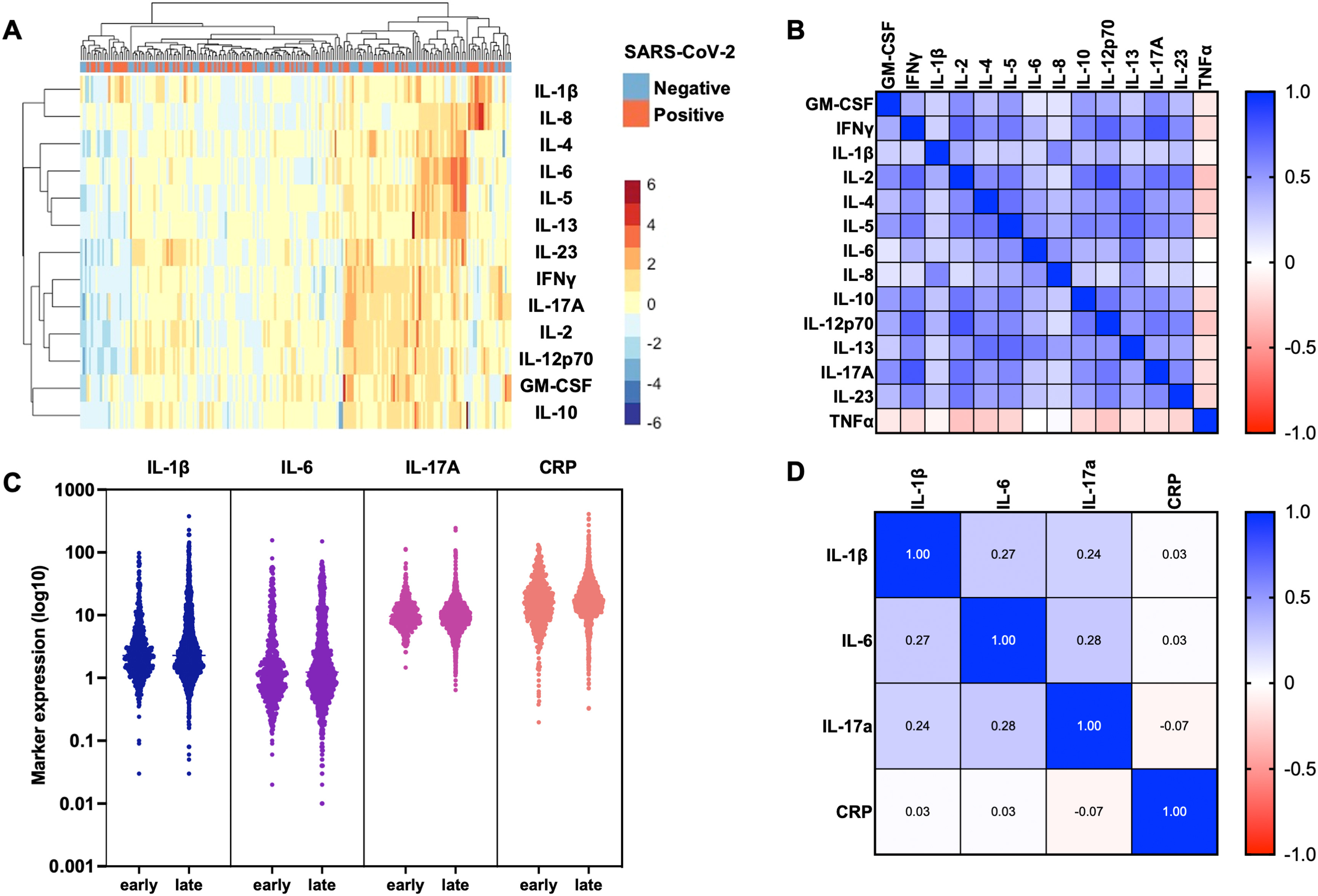

Supplement: Suppl figure 2 [file NIHMS1987502-supplement-Suppl_figure_2.jpg]
