# Supplementary material for: SARS-CoV-2 infection, inflammation and birth outcomes in a prospective NYC pregnancy cohort
Source: J Reprod Immunol. Author manuscript; Available in PMC 2024 Jun 2. (PMC11144074; doi:10.1016/j.jri.2024.104243)
Supplement: Suppl doc [file NIHMS1987502-supplement-Suppl_doc.docx]

**Supplementary Table 1.** Overview of existing literature on cytokine levels after SARS-CoV-2 infection during pregnancy. Sample sizes reflect the number of participants included in cytokine analyses. T = trimester.

| **Author, year** | **Sample size (Cytokine analysis)** | **Study design** | **Main findings (Cytokine analysis)** | **Timing of infection assessment** | **Timing of cytokine assessment** |
| --- | --- | --- | --- | --- | --- |
| Tanacan et al., 2021 (Tanacan et al., 2021) | 180 (Pregnant participants with SARS-CoV-2 infection, n=90; Pregnant, gestational age-matched participants with no SARS-CoV-2 infection, n=90). | Case-control | Increased IFN-γ in T3 in cases compared to controls. Increased IL-2 in T1 and T2, and increased IL-10 and IL-17 in T1 in controls compared to cases. IL-6 not significantly different. | Infection status determined through positive PCR test. Infected (n=30) vs controls (n=30) during each trimester. No distinction between asymptomatic and symptomatic. | On hospital admission. |
| Chen et al., 2021 (Chen et al., 2021) | 25 (Pregnant with SARS-CoV-2 infection, n=11; Non-pregnant with SARS-CoV-2 infection, n=4; Pregnant with no SARS-CoV-2 infection, n=10). | Retrospective | Decreased IL-2, IL-10 and IL-12p70 and increased IL-4, TNF-α, TNF-β and IL-9 in non-pregnant cases compared to controls. Decreased IL-12p40 and Il-12p70 in pregnant cases compared to controls. | Infection status determined through RNA or antibody test. Distinction between mild, severe, critical cases. | Cytokine assessment time unclear. No distinction between early/late gestation cytokine measurement. |
| Taglauer et al., 2022 (Taglauer et al., 2022) | 60 (Pregnant with SARS-CoV-2 infection, n=31; Pregnant with no SARS-CoV-2 infection, n=28). | Prospective cohort | Increased IL-6 and IL-8 in cases of early infection, but not late infection, compared to controls. | Infection status determined through positive PCR test. Majority infected during pregnancy (83.9%) of which 90% was symptomatic, 4 asymptomatic at delivery, 1 symptomatic at delivery. Distinction between early and late covid infection. | At delivery. |
| Sherer et al., 2021 (Sherer et al., 2021) | 50 (Pregnant with SARS-CoV-2 infection, n=22; Non-pregnant with SARS-CoV-2 infection, n=17; Pregnant with no SARS-CoV-2 infection, n=11) | Case-control | No difference in IL-1β and IL-6 mRNA expression between groups. Increased IL-1β mRNA expression in samples collected within 14 days of infection compared to after 14 days of infection. | Infection status determined through positive PCR test. No distinction between trimester of infection. No distinction between asymptomatic and symptomatic. | Convenience sampling throughout gestation. No distinction between early/late gestation cytokines. |
| Rosen et al., 2022 (Rosen et al., 2022) | 69 (Pregnant with SARS-CoV-2 infection, n=44; Pregnant controls, n=25). | Retrospective cohort | Increased IL-18, IL-1RA and IP-10 in cases compared to controls. | Infection status determined through screening for symptoms and PCR test upon admission. Distinction between early, middle, late gestation infection. Distinction between symptomatic and asymptomatic. | On hospital admission in 3rd trimester or at delivery. |
| Trilla et al., 2022 (Trilla et al., 2022) | 124 (Pregnant with asymptomatic SARS-CoV-2 infection, n=90; Pregnant with symptomatic SARS-CoV-2 infection, n=34) | Multicenter prospective cohort | No difference in IL-6 between asymptomatic and symptomatic participants. Leukocytes, lymphocytes and platelets were not different between controls (n=693) and cases (n=124). | Infection status determined based on positive PCR test at delivery. Distinction between asymptomatic and symptomatic infection. | On hospital admission. |
| Rubio et al., 2022 (Rubio et al., 2022) | 36 (Pregnant with SARS-CoV-2 infection, n=23 (12 asymptomatic, 11 symptomatic); Pregnant with no SARS-CoV-2 infection, n=13) | Prospective case-control | Increased EGF, MIG, MIP-1β IL-13, IL-2R, IL-17 and IP-10 in cases compared to controls. Increased EGF, FGF, HGF, MCP-1, MIG, G-CSF, IL-1β , IP-10 , TNF-α, IL-15, MIP-1α, MIP-1β, IL-2, IL-2R, and IL-6 in symptomatic compared to asymptomatic cases. | Infection determined based on PCR test in 3d trimester or seropositive just after delivery. | At delivery. |
| Garcia-Flores et al., 2022 (Garcia-Flores et al., 2021) | 23 (Pregnant with SARS-CoV-2 infection, n=12 (8 asymptomatic, 1 mild, 3 severe); Pregnant with no SARS-CoV-2 infection, n=11) | Case-control | Increased IL-8, IL-10, IL-15 in cases compared to controls. No difference in IL-6, Il-16, Il-17A, IFN-γ ad TNF between cases and controls. | Infection status determined through PCR at admission. | On hospital admission. |
| Brancaccio et al., 2022 (Brancaccio et al., 2022) | 44 (Pregnant with SARS-CoV-2 infection, n=22; Pregnant with no SARS-CoV-2 infection, n=22) | Case-control | Increased TNF-α, IL-2, TGF-β, IL-10 gene expression in cases compared to controls. Increased IL-6 and IL-8 serum levels in cases compared to controls. | Infection status determined through PCR at admission. | On hospital admission. |
| Gee et al., 2021 (Gee et al., 2021) | 29 (Pregnant with recent or ongoing SARS-CoV-2 infection, n=15; Pregnant recovered from SARS-CoV-2 infection, n=14) | Prospective cohort | Increased IL-1β in cases compared to controls. No difference in IL-10, CXCL8 and Il-6 levels between cases and controls. | Infection status determined through antibody testing. | At delivery. |
| Cerbulo-Vazquez et al., 2022 (Cérbulo-Vázquez et al., 2022) | 43 (Pregnant with SARS-CoV-2 infection, n=15; Non-pregnant with SARS-CoV-2 infection, n=15; Pregnant with no SARS-CoV-2 infection, n=13) | Case-control | Increased TNF-α and Ll-4 compared to pregnant controls and non-pregnant cases. Increased IL-6 compared to pregnant controls. | Infection status determined through PCR at inclusion. | At inclusion. |
| Boelig et al., 2022 (Boelig et al., 2022) | 242 (Pregnant with SARS-CoV-2 infection, n=58; Pregnant with no SARS-CoV-2 infection, n=142; Pregnant with COVID-19 vaccination, n=42) | Retrospective cohort | Increased IL-6 in cases compared to controls. | Infection status determined through PCR. | At delivery. |

**Supplementary Table 2.** Univariate and multivariate quantile regression of SARS-CoV-2 infection (independent variable) on dependent variables birthweight and gestational age at delivery, n=2,352.

|  | **Birthweight (grams)**  25% Quantile | | **Birthweight (grams)**  Median: 50% Quantile | | **Birthweight (grams)**  75% Quantile | |
| --- | --- | --- | --- | --- | --- | --- |
| **Infection group** | **Coefficient**  **(95% CI)** | **P-Value** | **Coefficient**  **(95% CI)** | **P-Value** | **Coefficient**  **(95% CI)** | **P-Value** |
| **Unadjusted Analysis** |  |  |  |  |  |  |
| *Not Infected (n=1,981)* | *Ref.* | *Ref.* | *Ref.* | *Ref.* | *Ref.* | *Ref.* |
| Early Gestation Infection (<20w) (n=98) | -19.84  (-151.83; 112.15) | 0.768 | 4.82  (-115.39; 125.03) | 0.937 | 29.76  (-99.83; 159.35) | 0.658 |
| Late Gestation Infection (≥20w) (n=273) | -74.84  (-157.18; 7.50) | 0.075 | -25.23  (-100.22; 49.76) | 0.509 | **-100.06**  **(-180.90; -19.22)** | **0.017** |
| *Not Infected (n=1,981)* | *Ref.* | *Ref.* | *Ref.* | *Ref.* | *Ref.* | *Ref.* |
| Infected Any Time During Gestation (n=371) | **-74.84**  **(-146.92; 2.14)** | **.042** | -15.02  (-79.71; 49.67) | 0.649 | -70.03  (-140.88; 0.82) | 0.053 |
| **Adjusted Analysis*** |  |  |  |  |  |  |
| *Not Infected (n=1,981)* | *Ref.* | *Ref.* | *Ref.* | *Ref.* | *Ref.* | *Ref.* |
| Early Gestation Infection (<20w) (n=98) | 69.27 (-41.17; 179.72) | 0.219 | **103.96 (2.73; 205.19)** | **0.044** | 107.13 (-17.11; 231.37) | 0.091 |
| Late Gestation Infection (≥20w) (n=273) | -41.28 (-106.17; 23.61) | 0.224 | -39.02 (-98.50; 20.46) | 0.198 | -38.21 (-111.21; 34.78) | 0.305 |
| *Not Infected (n=1,981)* | *Ref.* | *Ref.* | *Ref.* | *Ref.* | *Ref.* | *Ref.* |
| Infected Any Time During Gestation (n=371) | -3.79 (-62.42; 54.83) | 0.899 | -9.35 (-63.80; 45.10) | 0.736 | -3.41 (-69.92; 63.10) | 0.920 |
|  | **Gestational age at delivery (days)**  25% Quantile | | **Gestational age at delivery (days)**  Median: 50% Quantile | | **Gestational age at delivery (days)**  75% Quantile | |
| **Infection group** | **Coefficient**  **(95% CI)** | **P-Value** | **Coefficient**  **(95% CI)** | **P-Value** | **Coefficient**  **(95% CI)** | **P-Value** |
| **Unadjusted Analysis** |  |  |  |  |  |  |
| *Not Infected (n=1,981)* | *Ref.* | *Ref.* | *Ref.* | *Ref.* | *Ref.* | *Ref.* |
| Early Gestation Infection (<20w) (n=98) | -2.00 (-5.98; 1.98) | 0.325 | 0.00 (-1.48; 1.48) | >0.999 | 0.00 (-2.49; 2.49) | >0.999 |
| Late Gestation Infection (≥20w) (n=273) | -2.00 (-4.49; 0.49) | 0.115 | 0.00 (-0.92; 0.92) | >0.999 | -1.00 (-2.55; 0.55) | 0.207 |
| *Not Infected (n=1,981)* | *Ref.* | *Ref.* | *Ref.* | *Ref.* | *Ref.* | *Ref.* |
| Infected Any Time During Gestation (n=371) | -2.00 (-4.18; 0.18) | 0.072 | 0.00 (-0.81; 0.81) | >0.999 | -1.00 (-2.36; 0.36) | 0.150 |
| **Adjusted Analysis*** |  |  |  |  |  |  |
| *Not Infected (n=1,981)* | *Ref.* | *Ref.* | *Ref.* | *Ref.* | *Ref.* | *Ref.* |
| Early Gestation Infection (<20w) (n=98) | 0.67 (-2.41; 3.75) | 0.670 | 1.70 (-0.21; 3.62) | 0.082 | 1.81 (-0.22; 3.83) | 0.081 |
| Late Gestation Infection (≥20w) (n=273) | -0.66 (-2.47; 1.15) | 0.475 | 0.08 (-1.05; 1.21) | 0.888 | -0.03 (-1.22; 1.16) | 0.961 |
| *Not Infected (n=1,981)* | *Ref.* | *Ref.* | *Ref.* | *Ref.* | *Ref.* | *Ref.* |
| Infected Any Time During Gestation (n=371) | -0.32 (-1.95; 1.32) | 0.703 | 0.4347 (-0.59; 1.45) | 0.404 | 0.31 (-0.76; 1.39) | 0.565 |

Coefficients, CI and P-values in **bold** signify statistically significant results.

*Adjusted for maternal age (18-34, 35-49), race/ethnicity (Asian (non-Hispanic), Black (non-Hispanic), Hispanic, White (non-Hispanic), any other race), insurance (private/self-pay, public), parity (nulliparous, multiparous), pre-pregnancy BMI (underweight (<18.5) & normal weight (18.5-24.9), overweight (25.0-29.9), obese (>30)), vaccinated any one dose (yes during pregnancy, yes prior to pregnancy, no), history of preterm birth (yes, no), chronic hypertension (yes, no), pre-existing diabetes (yes, no), gestational hypertension (yes, no), gestational diabetes (yes, no), pre-eclampsia (yes, no), and time since start of pandemic (weeks). Birthweight analyses were also adjusted for gestational age at delivery and fetal sex (male, female).

**Supplementary Table 3.** Comparison of gestational age at birth (weeks) and birthweight (grams), as well as immune markers (IL-1$\beta$, IL-6, IL-17A, HS-CRP) between SARS-CoV-2 infected participants categorized per detection mode (Anti-S IgG, RT-PCR and both Anti-S IgG & RT-PCR) and never infected participants. Pregnancy outcomes nor immune markers were significantly different between groups.

|  | **Never infected**  **(n=1,981)** | **Anti-S IgG (n=207)** | **RT-PCR**  **(n=87)** | **Anti-S IgG & RT-PCR (n=74)** | **p-value** |
| --- | --- | --- | --- | --- | --- |
| **Pregnancy outcomes** | | | | | |
| Gestational age at birth (weeks) | 39.1 (1.6) | 39.3 (1.6) | 39.0 (2.0) | 39.0 (2.0) | 0.099 |
| Birthweight (grams) | 3255.1 (615.2) | 3250.0 (559.9) | 3210.0 (695.1) | 3224.9 (612.4) | 0.504 |
| **Cytokines & HS-CRP** | | | | | |
| IL-1$\beta$ | 2.3 (3.5) | 2.1 (3.5) | 1.4 (2.1) | 2.4 (3.2) | 0.764 |
| IL-6 | 1.2 (2.6) | 1.0 (3.7) | 1.0 (1.8) | 1.7 (3.9) | 0.214 |
| IL-17A | 9.6 (6.3) | 9.3 (7.0) | 10.4 (6.0) | 9.8 (5.5) | 0.835 |
| HS-CRP | 16.2 (14.1) | 20.8 (17.8) | 17.1 (24.9) | 20.5 (24.2) | 0.594 |

**Supplementary Table 4.** Univariate and multivariate quantile regression of early and late gestation immune markers on birthweight (grams) in a subgroup of infected participants, n=285.

|  | **Birthweight (grams)**  25% Quantile | | **Birthweight (grams)**  Median: 50% Quantile | | **Birthweight (grams)**  75% Quantile | |
| --- | --- | --- | --- | --- | --- | --- |
| **Early Gestation (<20w), n=54** | **Coefficient (95% CI)** | **P-Value** | **Coefficient (95% CI)** | **P-Value** | **Coefficient (95% CI)** | **P-Value** |
| **Unadjusted Analysis** |  |  |  |  |  |  |
| IL-1$\beta$ | **138.35 (11.55; 265.15)** | **0.033** | **119.63 (31.39; 207.86)** | **0.009** | **149.95 (16.24; 283.67)** | **0.029** |
| IL-6 | -41.16 (-114.22; 31.89) | 0.263 | **-71.74 (-141.5; -1.97)** | **0.044** | -75.15 (-175.25; 24.96) | 0.138 |
| IL-17A | -58.31 (-230.80; 114.19) | 0.500 | -39.31 (-229.94; 151.33) | 0.681 | -90.34 (-317.04; 136.36) | 0.427 |
| HS-CRP | -101.30 (-279.10; 76.49) | 0.258 | -122.66 (-279.85; 34.53) | 0.123 | 6.25 (-204.64; 217.14) | 0.953 |
| **Adjusted Analysis*** |  |  |  |  |  |  |
| IL-1$\beta$ | -29.75 (-66.78; 7.28) | 0.108 | 11.26 (-24.75; 47.26) | 0.517 | -17.99 (-69.08; 33.12) | 0.466 |
| IL-6 | **-46.85(-54.18; -39.52)** | **<0.001** | **-42.48 (-55.88; -29.07)** | **<0.001** | 16.82 (-2.25; 35.89) | 0.080 |
| IL-17A | **-72.37 (-141.54; -3.21)** | **0.041** | **-96.74 (-115.27; -78.21)** | **<0.001** | **-49.23 (-67.19; -31.27)** | **<0.001** |
| HS-CRP | **267.19 (134.81; 399.57)** | **<0.001** | **197.09 (131.19; 263.00)** | **<0.001** | **280.21 (72.03; 488.39)** | **0.011** |
|  | **Birthweight (grams)**  25% Quantile | | **Birthweight (grams)**  Median: 50% Quantile | | **Birthweight (grams)**  75% Quantile | |
| **Late Gestation (≥20w), n=231** | **Coefficient (95% CI)** | **P-Value** | **Coefficient (95% CI)** | **P-Value** | **Coefficient (95% CI)** | **P-Value** |
| **Unadjusted Analysis** |  |  |  |  |  |  |
| IL-1$\beta$ | -36.85 (-93.04; 19.34) | 0.198 | -14.43 (-57.60; 28.75) | 0.511 | -24.07 (-70.44; 22.31) | 0.308 |
| IL-6 | 1.61 (-40.85; 44.07) | 0.941 | 9.64 (-22.69; 41.98) | 0.557 | 17.14 (-15.82; 50.11) | 0.307 |
| IL-17A | 9.11 (-95.51; 113.72) | 0.864 | 60.35 (-20.92; 141.63) | 0.145 | 75.04 (-2.64; 152.73) | 0.058 |
| HS-CRP | -70.95 (-155.55; 13.66) | 0.100 | -41.97 (-114.82; 30.88) | 0.258 | -37.83 (-112.91; 37.25) | 0.322 |
| **Adjusted Analysis*** |  |  |  |  |  |  |
| IL-1$\beta$ | 5.48 (-24.58; 35.83) | 0.722 | -3.71 (-41.27; 33.85) | 0.846 | -1.73 (-47.13; 43.68) | 0.940 |
| IL-6 | 14.43 (-9.36; 38.21) | 0.233 | 15.85 (-14.00; 45.70) | 0.296 | 1.09 (-32.81; 34.98) | 0.950 |
| IL-17A | 46.18 (-6.78; 99.14) | 0.087 | **82.11 (20.23; 144.00)** | **0.010** | 52.80 (-20.17; 125.77) | 0.155 |
| HS-CRP | -27.16 (-76.63; 22.30) | 0.280 | -17.29 (-77.51; 42.93) | 0.572 | 5.54 (-66.66; 77.74) | 0.880 |

Coefficients, CI and P-values in **bold** signify statistically significant results. Immune markers are log2 transformed and analyzed as continuous variables.

*Adjusted for maternal age (18-34, 35-49), race/ethnicity (Asian (non-Hispanic), Black (non-Hispanic), Hispanic, White (non-Hispanic), any other race), insurance (private/self-pay, public), parity (nulliparous, multiparous), pre-pregnancy BMI (underweight (<18.5) & normal weight (18.5-24.9), overweight (25.0-29.9), obese (>30)), vaccinated any one dose (yes during pregnancy, yes prior to pregnancy, no), history of preterm birth (yes, no), chronic hypertension (yes, no), pre-existing diabetes (yes, no), gestational hypertension (yes, no), gestational diabetes (yes, no), pre-eclampsia (yes, no), time since start of pandemic, gestational age at delivery, fetal sex, multiplex assay batch, and gestational age at specimen collection.

**Supplementary Table 5.** Univariate and multivariate quantile regression of early and late gestation immune markers on gestational age at delivery (days) in a subgroup of infected participants, n=285.

|  | **Gestational age at delivery (days)**  25% Quantile | | **Gestational age at delivery (days)**  Median: 50% Quantile | | **Gestational age at delivery (days)**  75% Quantile | |
| --- | --- | --- | --- | --- | --- | --- |
| **Early Gestation (<20w), n=54** | **Coefficient (95% CI)** | **P-Value** | **Coefficient (95% CI)** | **P-Value** | **Coefficient (95% CI)** | **P-Value** |
| **Unadjusted Analysis** |  |  |  |  |  |  |
| IL-1$\beta$ | **3.64 (0.08; 7.21)** | **0.045** | 1.44 (-1.38; 4.25) | 0.310 | 1.11 (-0.91; 3.12) | 0.275 |
| IL-6 | 0.00 (-2.23; 2.23) | >0.999 | -0.20 (-1.67; 1.24) | 0.784 | 0.51 (-1.06; 2.08) | 0.518 |
| IL-17A | 1.04 (-4.24; 6.32) | 0.694 | -0.35 (-3.90; 3.19) | 0.843 | 1.96 (-1.92; 5.83) | 0.316 |
| HS-CRP | 0.43 (-5.48; 6.34) | 0.885 | 0.33 (-2.58; 3.25) | 0.821 | -1.24 (-4.15; 1.66) | 0.395 |
| **Adjusted Analysis*** |  |  |  |  |  |  |
| IL-1$\beta$ | -0.67 (-2.53; 1.20) | 0.461 | -0.06 (-2.20; 2.08) | 0.954 | -0.56 (-2.54; 1.42) | 0.557 |
| IL-6 | -0.02 (-1.22; 1.19) | 0.980 | 0.11 (-1.22; 1.43) | 0.866 | 1.22 (-2.18, 4.62) | 0.471 |
| IL-17A | -1.44 (-4.16; 1.29 | 0.285 | 0.27 (-1.54; 2.08) | 0.758 | 2.00 (-0.27; 4.28) | 0.081 |
| HS-CRP | 4.10 (-1.61; 9.81) | 0.150 | 0.03 (-5.33, 5.38) | 0.992 | 2.74 (-2.02; 7.51) | 0.247 |
|  | **Gestational age at delivery (days)**  25% Quantile | | **Gestational age at delivery (days)**  Median: 50% Quantile | | **Gestational age at delivery (days)**  75% Quantile | |
| **Late Gestation (≥20w), n=231** | **Coefficient (95% CI)** | **P-Value** | **Coefficient (95% CI)** | **P-Value** | **Coefficient (95% CI)** | **P-Value** |
| **Unadjusted Analysis** |  |  |  |  |  |  |
| IL-1$\beta$ | -0.95 (-2.49; 0.59) | 0.226 | 0.00 (-0.71; 0.71) | >0.999 | -0.24 (-1.11; 0.63) | 0.592 |
| IL-6 | 0.35 (-0.82; 1.52) | 0.558 | 0.00 (-0.69, 0.69) | >0.999 | 0.00 (-0.65; 0.65) | >0.999 |
| IL-17A | 1.12 (-1.76; 3.99) | 0.444 | -0.30 (-1.73; 1.14) | 0.686 | 0.74 (-0.76; 2.25) | 0.332 |
| HS-CRP | **-2.67 (-4.95; -0.40)** | **0.021** | -0.66 (-1.95; 0.62) | 0.309 | -0.67 (-2.06; 0.71) | 0.338 |
| **Adjusted Analysis*** |  |  |  |  |  |  |
| IL-1$\beta$ | **-1.22 (-2.00; -0.43)** | **0.003** | -0.52 (-1.13; 0.278) | 0.088 | **-0.63 (-1.13; -0.13)** | **0.014** |
| IL-6 | -0.25 (-0.97; 0.46) | 0.483 | -0.37 (-0.84; 0.10) | 0.126 | -0.17(-0.64; 0.31) | 0.485 |
| IL-17A | -1.56 (-3.20; 0.07) | 0.060 | -0.94 (-1.94; 0.06) | 0.066 | -0.86 (-1.94; 0.21) | 0.115 |
| HS-CRP | -0.94 (-2.64; 0.75) | 0.274 | -0.53 (-1.53; 0.48) | 0.301 | -0.37 (-1.28; 0.55) | 0.428 |

Coefficients, CI and P-values in **bold** signify statistically significant results. Immune markers are log2 transformed and analyzed as continuous variables.

*Adjusted for maternal age (18-34, 35-49), race/ethnicity (Asian (non-Hispanic), Black (non-Hispanic), Hispanic, White (non-Hispanic), any other race), insurance (private/self-pay, public), parity (nulliparous, multiparous), pre-pregnancy BMI (underweight (<18.5) & normal weight (18.5-24.9), overweight (25.0-29.9), obese (>30)), vaccinated any one dose (yes during pregnancy, yes prior to pregnancy, no), history of preterm birth (yes, no), chronic hypertension (yes, no), pre-existing diabetes (yes, no), gestational hypertension (yes, no), gestational diabetes (yes, no), pre-eclampsia (yes, no), time since start of pandemic, multiplex assay batch, and gestational age at specimen collection.

**Supplementary Table 6a.** Univariate and multivariate logistic regression of early gestation (<20w) SARS-CoV-2 infection on early gestation high HS-CRP (>40mg/L)**

|  | **Early gestation high HS-CRP** | |
| --- | --- | --- |
| **Infection group** | **Coefficient (95% CI)** | **P-Value** |
| **Unadjusted Analysis** |  |  |
| Not Infected (N=491) | Ref. | Ref. |
| Early Gestation Infection (<20w) (N=54) | 1.65 (0.81; 3.36) | 0.167 |
| **Adjusted Analysis*** |  |  |
| Not Infected (N=491) | Ref. | Ref. |
| Early Gestation Infection (<20w) (N=54) | 0.84 (0.31; 2.33) | 0.842 |

Coefficients, CI and P-values in **bold** signify statistically significant results.

*Adjusted for maternal age (18-34, 35-49), race/ethnicity (Asian (non-Hispanic), Black (non-Hispanic), Hispanic, White (non-Hispanic), any other race), insurance (private/self-pay, public), parity (nulliparous, multiparous), pre-pregnancy BMI (underweight (<18.5) & normal weight (18.5-24.9), overweight (25.0-29.9), obese (>30)), vaccinated any one dose (yes during pregnancy, yes prior to pregnancy, no), history of preterm birth (yes, no), chronic hypertension (yes, no), pre-existing diabetes (yes, no), gestational hypertension (yes, no), gestational diabetes (yes, no), pre-eclampsia (yes, no), time since start of pandemic, multiplex assay batch, and gestational age at specimen collection.

** Sample size adjusted to N=545 based on specimen availability. Early gestation infection N=54 (high HS-CRP n=11; normal HS-CRP n=43) and not infected N=491 (high HS-CRP n=66, normal HS-CRP n=425).

**Supplementary Table 6b**. Univariate and multivariate logistic regression of early gestation SARS-CoV-2 infection on late gestation high HS-CRP (>40mg/L)**

|  | **Late gestation high HS-CRP** | |
| --- | --- | --- |
| **Infection group** | **Coefficient (95% CI)** | **P-Value** |
| **Unadjusted Analysis** |  |  |
| Not Infected (N=1318) | Ref. | Ref. |
| Early Gestation Infection (<20w) (N=30) | 0.69 (0.16; 2.93) | 0.613 |
| **Adjusted Analysis*** |  |  |
| Not Infected (N=1318) | Ref. | Ref. |
| Early Gestation Infection (<20w) (N=30) | 0.49 (0.11, 2.21) | 0.351 |

Coefficients, CI and P-values in **bold** signify statistically significant results.

*Adjusted for maternal age (18-34, 35-49), race/ethnicity (Asian (non-Hispanic), Black (non-Hispanic), Hispanic, White (non-Hispanic), any other race), insurance (private/self-pay, public), parity (nulliparous, multiparous), pre-pregnancy BMI (underweight (<18.5) & normal weight (18.5-24.9), overweight (25.0-29.9), obese (>30)), vaccinated any one dose (yes during pregnancy, yes prior to pregnancy, no), history of preterm birth (yes, no), chronic hypertension (yes, no), pre-existing diabetes (yes, no), gestational hypertension (yes, no), gestational diabetes (yes, no), pre-eclampsia (yes, no), time since start of pandemic, multiplex assay batch, and gestational age at specimen collection.

** Sample size adjusted to N=1348 based on specimen availability. Early gestation infection N=30 (high HS-CRP n=2, normal HS-CRP n=28) and not infected N=1318 (high HS-CRP n=124, normal HS-CRP n=1194).

**Supplementary Table 6c**. Univariate and multivariate logistic regression of late gestation (≥20w) SARS-CoV-2 infection on late gestation high HS-CRP (>40mg/L)**

|  | **Late gestation high HS-CRP** | |
| --- | --- | --- |
| **Infection group** | **Coefficient (95% CI)** | **P-Value** |
| **Unadjusted Analysis** |  |  |
| Not Infected (N=1318) | Ref. | Ref. |
| Late Gestation Infection (≥20w) (N=230) | **2.04 (1.36; 3.08)** | **<0.001** |
| **Adjusted Analysis*** |  |  |
| Not Infected (N=1318) | Ref. | Ref. |
| Late Gestation Infection (≥20w) (N=230) | 1.49 (0.94; 2.36) | 0.088 |

Coefficients, CI and P-values in **bold** signify statistically significant results.

*Adjusted for maternal age (18-34, 35-49), race/ethnicity (Asian (non-Hispanic), Black (non-Hispanic), Hispanic, White (non-Hispanic), any other race), insurance (private/self-pay, public), parity (nulliparous, multiparous), pre-pregnancy BMI (underweight (<18.5) & normal weight (18.5-24.9), overweight (25.0-29.9), obese (>30)), vaccinated any one dose (yes during pregnancy, yes prior to pregnancy, no), history of preterm birth (yes, no), chronic hypertension (yes, no), pre-existing diabetes (yes, no), gestational hypertension (yes, no), gestational diabetes (yes, no), pre-eclampsia (yes, no), time since start of pandemic, multiplex assay batch, and gestational age at specimen collection.

** Sample size adjusted to N=1548 based on specimen availability. Early gestation infection N=230 (high HS-CRP n=37, normal HS-CRP n=193) and not infected N=1318 (high HS-CRP n=124, normal HS-CRP n=1194).

**Supplementary Table 7a.** Univariate and multivariate quantile regression of early and late gestation high HS-CRP (>40mg/L) on birthweight (grams) compared to a reference group of normal HS-CRP, n=2,101.

|  | **Birthweight (grams)**  **25% Quantile** | | **Birthweight (grams)**  **50% Quantile (Median)** | | **Birthweight (grams)**  **75% Quantile** | |
| --- | --- | --- | --- | --- | --- | --- |
| **Early Gestation (<20w), n=547** | **Coefficient (95% CI)** | **P-Value** | **Coefficient (95% CI)** | **P-Value** | **Coefficient (95% CI)** | **P-Value** |
| **Unadjusted Analysis** |  |  |  |  |  |  |
| Normal HS-CRP (<20w) (n=469) | Ref. | Ref. | Ref. | Ref. | Ref. | Ref. |
| High HS-CRP (<20w) (n=77) | -39.98 (-221.36; 141.40) | 0.665 | 5.10 (-132.94; 143.14) | 0.942 | -70.02 (-235.96; 95.92) | 0.408 |
| **Adjusted Analysis*** |  |  |  |  |  |  |
| Normal HS-CRP (<20w) (n=469) | Ref. | Ref. | Ref. | Ref. | Ref. | Ref. |
| High HS-CRP (<20w) (n=77) | 40.12 (-92.97; 173.21) | 0.554 | -19.41 (-139.79; 100.96) | 0.751 | 89.48 (-58.77; 237.72) | 0.236 |
|  | **Birthweight (grams)**  **25% Quantile** |  | **Birthweight (grams)**  **50% Quantile (Median)** |  | **Birthweight (grams)**  **75% Quantile** |  |
| **Late Gestation (≥20w), n=1,554** | **Coefficient (95% CI)** | **P-Value** | **Coefficient (95% CI)** | **P-Value** | **Coefficient (95% CI)** | **P-Value** |
| **Unadjusted Analysis** |  |  |  |  |  |  |
| Normal HS-CRP (≥20w) (n=1,388) | Ref. | Ref. | Ref. | Ref. | Ref. | Ref. |
| High HS-CRP (≥20w) (n=161) | **-190.22 (-289.79; -90.65)** | **<0.001** | **-105.18 (-202.39; -7.98)** | **0.034** | -74.84 (-185.49, 35.81) | 0.185 |
| **Adjusted Analysis*** |  |  |  |  |  |  |
| Normal HS-CRP (≥20w) (n=1,388) | Ref. | Ref. | Ref. | Ref. | Ref. | Ref. |
| High HS-CRP (≥20w) (n=161) | -62.44 (-145.75; 20.88) | 0.142 | -58.47 (-138.20; 21.27) | 0.151 | -9.46 (-110.49; 91.56) | 0.854 |

Coefficients, CI and P-values in **bold** signify statistically significant results.

*Adjusted for maternal age (18-34, 35-49), race/ethnicity (Asian (non-Hispanic), Black (non-Hispanic), Hispanic, White (non-Hispanic), any other race), insurance (private/self-pay, public), parity (nulliparous, multiparous), pre-pregnancy BMI (underweight (<18.5) & normal weight (18.5-24.9), overweight (25.0-29.9), obese (>30)), vaccinated any one dose (yes during pregnancy, yes prior to pregnancy, no), history of preterm birth (yes, no), chronic hypertension (yes, no), pre-existing diabetes (yes, no), gestational hypertension (yes, no), gestational diabetes (yes, no), pre-eclampsia (yes, no), time since start of pandemic, gestational age at delivery, fetal sex (male, female), multiplex assay batch, and gestational age at specimen collection.

**Supplementary Table 7b.** Univariate and multivariate quantile regression of early and late gestation high HS-CRP (>40mg/L) on gestational age at delivery (days) compared to a reference group of normal HS-CRP, n=2,101.

|  | **Gestational age at delivery (days)**  **25% Quantile** | | **Gestational age at delivery (days)**  **50% Quantile (Median)** | | **Gestational age at delivery (days)**  **75% Quantile** | |
| --- | --- | --- | --- | --- | --- | --- |
| **Early Gestation (<20w), n=547** | **Coefficient (95% CI)** | **P-Value** | **Coefficient (95% CI)** | **P-Value** | **Coefficient (95% CI)** | **P-Value** |
| **Unadjusted Analysis** |  |  |  |  |  |  |
| Normal HS-CRP (<20w) (n=469) | Ref. | Ref. | Ref. | Ref. | Ref. | Ref. |
| High HS-CRP (<20w) (n=77) | -4.00 (-9.31; 1.31) | 0.139 | -1.00 (-3.30; 1.30) | 0.394 | **-4.00 (-6.66, -1.35)** | **0.003** |
| **Adjusted Analysis*** |  |  |  |  |  |  |
| Normal HS-CRP (<20w) (n=469) | Ref. | Ref. | Ref. | Ref. | Ref. | Ref. |
| High HS-CRP (<20w) (n=77) | -1.02 (-4.80; 2.76) | 0.597 | -0.69 (-3.10; 1.73) | 0.577 | -0.01 (-2.38;2.37) | 0.995 |
|  | **Gestational age at delivery (days)**  **25% Quantile** | | **Gestational age at delivery (days)**  **50% Quantile (Median)** | | **Gestational age at delivery (days)**  **75% Quantile** | |
| **Late Gestation (≥20w), n=1,554** | **Coefficient (95% CI)** | **P-Value** | **Coefficient (95% CI)** | **P-Value** | **Coefficient (95% CI)** | **P-Value** |
| **Unadjusted Analysis** |  |  |  |  |  |  |
| Normal HS-CRP (≥20w) (n=1,388) | Ref. | Ref. | Ref. | Ref. | Ref. | Ref. |
| High HS-CRP (≥20w) (n=161) | **-7.00 (-10.32, -3.68)** | **<0.001** | -1.00 (-2.37; 0.37) | 0.152 | **-3.00 (-4.84; -1.16)** | **0.001** |
| **Adjusted Analysis*** |  |  |  |  |  |  |
| Normal HS-CRP (≥20w) (n=1,388) | Ref. | Ref. | Ref. | Ref. | Ref. | Ref. |
| High HS-CRP (≥20w) (n=161) | -1.90 (-4.20; 0.40) | 0.105 | **-2.65 (-4.08; -1.22)** | **<.001** | **-2.12 (-3.75; -0.49)** | **0.011** |

Coefficients, CI and P-values in **bold** signify statistically significant results.

*Adjusted for maternal age (18-34, 35-49), race/ethnicity (Asian (non-Hispanic), Black (non-Hispanic), Hispanic, White (non-Hispanic), any other race), insurance (private/self-pay, public), parity (nulliparous, multiparous), pre-pregnancy BMI (underweight (<18.5) & normal weight (18.5-24.9), overweight (25.0-29.9), obese (>30)), vaccinated any one dose (yes during pregnancy, yes prior to pregnancy, no), history of preterm birth (yes, no), chronic hypertension (yes, no), pre-existing diabetes (yes, no), gestational hypertension (yes, no), gestational diabetes (yes, no), pre-eclampsia (yes, no), time since start of pandemic, multiplex assay batch, and gestational age at specimen collection.

**Supplementary Table 8a.** Univariate and multivariate quantile regression of early and late gestation high HS-CRP (>40mg/L) on birthweight (grams) compared to a reference group of normal HS-CRP in a subgroup of infected participants, n=285.

|  | **Birthweight (grams)**  **25% Quantile** | | **Birthweight (grams)**  **50% Quantile (Median)** | | **Birthweight (grams)**  **75% Quantile** | |
| --- | --- | --- | --- | --- | --- | --- |
| **Early Gestation (<20w), n=54** | **Coefficient (95% CI)** | **P-Value** | **Coefficient (95% CI)** | **P-Value** | **Coefficient (95% CI)** | **P-Value** |
| **Unadjusted Analysis** |  |  |  |  |  |  |
| Normal HS-CRP (<20w) (n=43) | Ref. | Ref. | Ref. | Ref. | Ref. | Ref. |
| High HS-CRP (<20w) (n=11) | -189.94 (-678.82; 298.94) | 0.439 | -269.89 (-670.90; 131.12) | 0.183 | -139.76 (-663.67; 384.15) | 0.595 |
| **Adjusted Analysis*** |  |  |  |  |  |  |
| Normal HS-CRP (<20w) (n=43) | Ref. | Ref. | Ref. | Ref. | Ref. | Ref. |
| High HS-CRP (<20w) (n=11) | 175.40 (-90.35; 441.15) | 0.182 | 128.39 (-15.64; 272.42) | 0.077 | 371.35 (336.49; 406.21) | 0.303 |
|  | **Birthweight (grams)**  **25% Quantile** |  | **Birthweight (grams)**  **50% Quantile (Median)** |  | **Birthweight (grams)**  **75% Quantile** |  |
| **Late Gestation (≥20w), n=231** | **Coefficient (95% CI)** | **P-Value** | **Coefficient (95% CI)** | **P-Value** | **Coefficient (95% CI)** | **P-Value** |
| **Unadjusted Analysis** |  |  |  |  |  |  |
| Normal HS-CRP (≥20w) (n=193) | Ref. | Ref. | Ref. | Ref. | Ref. | Ref. |
| High HS-CRP (≥20w) (n=37) | -250.05 (-506.24; 88.34) | 0.056 | -145.15 (-343.67; 53.37) | 0.151 | -159.89 (-375.28; 55.50) | 0.145 |
| **Adjusted Analysis*** |  |  |  |  |  |  |
| Normal HS-CRP (≥20w) (n=193) | Ref. | Ref. | Ref. | Ref. | Ref. | Ref. |
| High HS-CRP (≥20w) (n=37) | -74.27 (-211.40; 62.86) | 0.287 | -110.20 (-259.99; 39.59) | 0.148 | -82.48 (-255.17; 90.20) | 0.347 |

Coefficients, CI and P-values in **bold** signify statistically significant results.

*Adjusted for maternal age (18-34, 35-49), race/ethnicity (Asian (non-Hispanic), Black (non-Hispanic), Hispanic, White (non-Hispanic), any other race), insurance (private/self-pay, public), parity (nulliparous, multiparous), pre-pregnancy BMI (underweight (<18.5) & normal weight (18.5-24.9), overweight (25.0-29.9), obese (>30)), vaccinated any one dose (yes during pregnancy, yes prior to pregnancy, no), history of preterm birth (yes, no), chronic hypertension (yes, no), pre-existing diabetes (yes, no), gestational hypertension (yes, no), gestational diabetes (yes, no), pre-eclampsia (yes, no), time since start of pandemic, gestational age at delivery, fetal sex (male, female), multiplex assay batch, and gestational age at specimen collection.

**Supplementary Table 8b.** Univariate and multivariate quantile regression of early and late gestation high HS-CRP (>40mg/L) on gestational age at delivery (days) compared to a reference group of normal HS-CRP in a subgroup of infected participants, n=285.

|  | **Gestational age at delivery (days)**  **25% Quantile** | | **Gestational age at delivery (days)**  **50% Quantile (Median)** | | **Gestational age at delivery (days)**  **75% Quantile** | |
| --- | --- | --- | --- | --- | --- | --- |
| **Early Gestation (<20w), n=54** | **Coefficient (95% CI)** | **P-Value** | **Coefficient (95% CI)** | **P-Value** | **Coefficient (95% CI)** | **P-Value** |
| **Unadjusted Analysis** |  |  |  |  |  |  |
| Normal HS-CRP (<20w) (n=43) | Ref. | Ref. | Ref. | Ref. | Ref. | Ref. |
| High HS-CRP (<20w) (n=11) | 4.00 (-12.42; 20.42) | 0.627 | 3.00 (-4.56; 10.56) | 0.429 | -2.00 (-9.82; 5.82) | 0.610 |
| **Adjusted Analysis*** |  |  |  |  |  |  |
| Normal HS-CRP (<20w) (n=43) | Ref. | Ref. | Ref. | Ref. | Ref. | Ref. |
| High HS-CRP (<20w) (n=11) | 6.81 (-3.91; 17.52) | 0.200 | **5.84 (1.92;9.76)** | **0.006** | -2.42 (-8.52; 3.68) | 0.418 |
|  | **Gestational age at delivery (days)**  **25% Quantile** |  | **Gestational age at delivery (days)**  **50% Quantile (Median)** |  | **Gestational age at delivery (days)**  **75% Quantile** |  |
| **Late Gestation (≥20w), n=231** | **Coefficient (95% CI)** | **P-Value** | **Coefficient (95% CI)** | **P-Value** | **Coefficient (95% CI)** | **P-Value** |
| **Unadjusted Analysis** |  |  |  |  |  |  |
| Normal HS-CRP (≥20w) (n=193) | Ref. | Ref. | Ref. | Ref. | Ref. | Ref. |
| High HS-CRP (≥20w) (n=37) | **-9.00 (-15.50, -2.50)** | **0.007** | -4.00 (-10.17, 2.17) | 0.203 | **-3.00 (-5.90, -0.10)** | **0.043** |
| **Adjusted Analysis*** |  |  |  |  |  |  |
| Normal HS-CRP (≥20w) (n=193) | Ref. | Ref. | Ref. | Ref. | Ref. | Ref. |
| High HS-CRP (≥20w) (n=37) | **-4.40 (-7.72; -1.08)** | **0.010** | **-3.49 (-6.00; -0.98)** | **0.007** | -2.10 (-4.84; 0.64) | 0.132 |

Coefficients, CI and P-values in **bold** signify statistically significant results.

*Adjusted for maternal age (18-34, 35-49), race/ethnicity (Asian (non-Hispanic), Black (non-Hispanic), Hispanic, White (non-Hispanic), any other race), insurance (private/self-pay, public), parity (nulliparous, multiparous), pre-pregnancy BMI (underweight (<18.5) & normal weight (18.5-24.9), overweight (25.0-29.9), obese (>30)), vaccinated any one dose (yes during pregnancy, yes prior to pregnancy, no), history of preterm birth (yes, no), chronic hypertension (yes, no), pre-existing diabetes (yes, no), gestational hypertension (yes, no), gestational diabetes (yes, no), pre-eclampsia (yes, no), time since start of pandemic, multiplex assay batch, and gestational age at specimen collection.

**References**

Boelig, R. C., Aghai, Z. H., Chaudhury, S., Kazan, A. S., Chan, J. S. Y., & Bergmann-Leitner, E. (2022). Impact of COVID-19 disease and COVID-19 vaccination on maternal or fetal inflammatory response, placental pathology, and perinatal outcomes. *American Journal of Obstetrics and Gynecology*, *227*(4), 652–656. https://doi.org/10.1016/j.ajog.2022.05.049

Brancaccio, M., Mennitti, C., Calvanese, M., Gentile, A., Musto, R., Gaudiello, G., Scamardella, G., Terracciano, D., Frisso, G., Pero, R., Sarno, L., Guida, M., & Scudiero, O. (2022). Diagnostic and Therapeutic Potential for HNP-1, HBD-1 and HBD-4 in Pregnant Women with COVID-19. *International Journal of Molecular Sciences*, *23*(7), 3450. https://doi.org/10.3390/ijms23073450

Cérbulo-Vázquez, A., García-Espinosa, M., Briones-Garduño, J. C., Arriaga-Pizano, L., Ferat-Osorio, E., Zavala-Barrios, B., Cabrera-Rivera, G. L., Miranda-Cruz, P., García de la Rosa, M. T., Prieto-Chávez, J. L., Rivero-Arredondo, V., Madera-Sandoval, R. L., Cruz-Cruz, A., Salazar-Rios, E., Salazar-Rios, M. E., Serrano-Molina, D., De Lira-Barraza, R. C., Villanueva-Compean, A. H., Esquivel-Pineda, A., … López-Macías, C. (2022). The percentage of CD39+ monocytes is higher in pregnant COVID-19+ patients than in nonpregnant COVID-19+ patients. *PLoS ONE*, *17*(7), e0264566. https://doi.org/10.1371/journal.pone.0264566

Chen, G., Liao, Q., Ai, J., Yang, B., Bai, H., Chen, J., Liu, F., Cao, Y., Liu, H., & Li, K. (2021). Immune Response to COVID-19 During Pregnancy. *Frontiers in Immunology*, *12*, 675476. https://doi.org/10.3389/fimmu.2021.675476

Garcia-Flores, V., Romero, R., Xu, Y., Theis, K., Arenas-Hernandez, M., Miller, D., Peyvandipour, A., Galaz, J., Levenson, D., Bhatti, G., Gershater, M., Pusod, E., Kracht, D., Florova, V., Leng, Y., Tao, L., Faucett, M., Para, R., Hsu, C.-D., … Gomez-Lopez, N. (2021). Maternal-Fetal Immune Responses in Pregnant Women Infected with SARS-CoV-2. *Research Square*, rs.3.rs-362886. https://doi.org/10.21203/rs.3.rs-362886/v1

Gee, S., Chandiramani, M., Seow, J., Pollock, E., Modestini, C., Das, A., Tree, T., Doores, K. J., Tribe, R. M., & Gibbons, D. L. (2021). The legacy of maternal SARS-CoV-2 infection on the immunology of the neonate. *Nature Immunology*, *22*(12), Article 12. https://doi.org/10.1038/s41590-021-01049-2

Rosen, D. B., Murphy, E. A., Gejman, R. S., Capili, A., Friedlander, R. L., Rand, S., Cagino, K. A., Glynn, S. M., Matthews, K. C., Kubiak, J. M., Yee, J., Prabhu, M., Riley, L. E., & Yang, Y. J. (2022). Cytokine response over the course of COVID-19 infection in pregnant women. *Cytokine*, *154*, 155894. https://doi.org/10.1016/j.cyto.2022.155894

Rubio, R., Aguilar, R., Bustamante, M., Muñoz, E., Vázquez-Santiago, M., Santano, R., Vidal, M., Melero, N. R., Parras, D., Serra, P., Santamaria, P., Carolis, C., Izquierdo, L., Gómez-Roig, M. D., Dobaño, C., Moncunill, G., & Mazarico, E. (2022). Maternal and neonatal immune response to SARS-CoV-2, IgG transplacental transfer and cytokine profile. *Frontiers in Immunology*, *13*, 999136. https://doi.org/10.3389/fimmu.2022.999136

Sherer, M. L., Lei, J., Creisher, P. S., Jang, M., Reddy, R., Voegtline, K., Olson, S., Littlefield, K., Park, H.-S., Ursin, R. L., Ganesan, A., Boyer, T., Elsayed, N., Brown, D. M., Walch, S. N., Antar, A. A. R., Manabe, Y. C., Jones-Beatty, K., Golden, W. C., … Burd, I. (2021). Pregnancy alters interleukin-1 beta expression and antiviral antibody responses during severe acute respiratory syndrome coronavirus 2 infection. *American Journal of Obstetrics and Gynecology*, *225*(3), 301.e1-301.e14. https://doi.org/10.1016/j.ajog.2021.03.028

Taglauer, E. S., Dhole, Y., Boateng, J., Snyder-Cappione, J., Parker, S. E., Clarke, K., Juttukonda, L., Devera, J., Hunnewell, J., Barnett, E., Jia, H., Yarrington, C., Sabharwal, V., & Wachman, E. M. (2022). Evaluation of maternal-infant dyad inflammatory cytokines in pregnancies affected by maternal SARS-CoV-2 infection in early and late gestation. *Journal of Perinatology: Official Journal of the California Perinatal Association*, *42*(10), 1319–1327. https://doi.org/10.1038/s41372-022-01391-9

Tanacan, A., Yazihan, N., Erol, S. A., Anuk, A. T., Yucel Yetiskin, F. D., Biriken, D., Ozgu-Erdinc, A. S., Keskin, H. L., Moraloglu Tekin, O., & Sahin, D. (2021). The impact of COVID-19 infection on the cytokine profile of pregnant women: A prospective case-control study. *Cytokine*, *140*, 155431. https://doi.org/10.1016/j.cyto.2021.155431

Trilla, C., Mora, J., Crovetto, F., Crispi, F., Gratacos, E., Llurba, E., & KidsCorona Pregnancy COVID-19 Group. (2022). First-Trimester SARS-CoV-2 Infection: Clinical Presentation, Inflammatory Markers, and Obstetric Outcomes. *Fetal Diagnosis and Therapy*, *49*(3), 67–76. https://doi.org/10.1159/000523974
